# Proteotranscriptomics assisted gene annotation and spatial proteomics of *Bombyx mori* BmN4 cell line

**DOI:** 10.1186/s12864-020-07088-7

**Published:** 2020-10-06

**Authors:** Michal Levin, Marion Scheibe, Falk Butter

**Affiliations:** Institute of Molecular Biology (IMB), Ackermannweg 4, 55128 Mainz, Germany

**Keywords:** Proteotranscriptomics, Mass spectrometry, Gene assembly, Gene annotation, Spatial proteomics

## Abstract

**Background:**

The process of identifying all coding regions in a genome is crucial for any study at the level of molecular biology, ranging from single-gene cloning to genome-wide measurements using RNA-seq or mass spectrometry. While satisfactory annotation has been made feasible for well-studied model organisms through great efforts of big consortia, for most systems this kind of data is either absent or not adequately precise.

**Results:**

Combining in-depth transcriptome sequencing and high resolution mass spectrometry, we here use proteotranscriptomics to improve gene annotation of protein-coding genes in the *Bombyx mori* cell line BmN4 which is an increasingly used tool for the analysis of piRNA biogenesis and function*.* Using this approach we provide the exact coding sequence and evidence for more than 6200 genes on the protein level. Furthermore using spatial proteomics, we establish the subcellular localization of thousands of these proteins. We show that our approach outperforms current *Bombyx mori* annotation attempts in terms of accuracy and coverage.

**Conclusions:**

We show that proteotranscriptomics is an efficient, cost-effective and accurate approach to improve previous annotations or generate new gene models. As this technique is based on de-novo transcriptome assembly, it provides the possibility to study any species also in the absence of genome sequence information for which proteogenomics would be impossible.

## Background

*Bombyx mori* was the first lepidopteran species whose draft genome was published in 2004 [1, 2]. In 2008, a more accurate genome assembly was generated by combining the raw data of these initial efforts within an international collaboration [3]⁠, and the results are available at SilkDB (www.silkdb.org) and KAIKObase (sgp.dna.affrc.go.jp/index.html). However, for a large number of modern applications such as transcriptomic, epigenomic and proteomic studies, reverse genetic screens and genome editing tools such as TALEN and CRISPR/Cas9 the provided genome information is insufficient as this assembly contains numerous non-sequenced chromosome regions. Recently, parallel to our efforts to reannotate *Bombyx mori* using proteotranscriptomics, two new initiatives provided improved genome assemblies. These new assemblies have been made available as SilkBase [4] and SilkDB 3.0 [5] and include more genomic regions and gene predictions for 16,880 and 16,069 gene models, receptively. However, the provided gene models are still based on automated gene prediction using limited full-length cDNA libraries, poly-A RNA-seq data and previous *B. mori* NCBI annotations. These predictions are made with a mixture of data from various commercial and non-commercial strains of *Bombyx mori,* thus may not represent the genomic sequence of a single strain or its derived cell line due to intraspecies genetic variation. Furthermore, only very few predicted *Bombyx mori* genes have evidence at the protein level (roughly 150 genes in UniProt UP000005204). Hence, an improved strain-specific gene annotation would likely improve global analyses.

*Bombyx mori* is similar to humans in terms of sensitivities to pathogens and comparable effects of drugs. The advantages for research are the low cost of maintenance, little ethical constraints and no biohazard risks. Hence, it has been long recognized as an excellent system for drug screening and safety assessment [5, 6]⁠. Furthermore, the BmN4 cell line of *Bombyx mori* [7]⁠ has been intensely used in studying many different biological aspects in the laboratory, including virus infection [8, 9]⁠ and germline piRNA biology [10]⁠. Despite the widespread usage of this ovary-derived cell line, its exact genomic sequence and a tested gene structure model is still missing. Natural sequence variations will interfere with primer design for gene amplification, the design of CRISPR guides and limit matching of short sequencing reads in transcriptomics as well as peptide coverage in proteomic experiments.

By proteotranscriptomics, combining in-depth transcriptome sequencing and high-resolution mass spectrometry, we establish protein evidence for 6273 genes. Using spatial proteomics, we additionally experimentally classify the localization of several thousand proteins using the recently published LOPIT-DC workflow [11], which utilizes differential ultracentrifugation following removal of unlysed cells to achieve enrichment of cellular compartments in different fractions. With this strategy, not only accurate gene models for the protein-coding genes, but also their subcellular localization is made available. Overall, we here provide a proof of concept for the generation of species-, strain- and cell line-specific gene annotation for protein-coding genes based on experimental evidence without the need of a sequenced genome.

## Results

### Transcriptome assembly from RNA-seq data

**Fig. 1:**
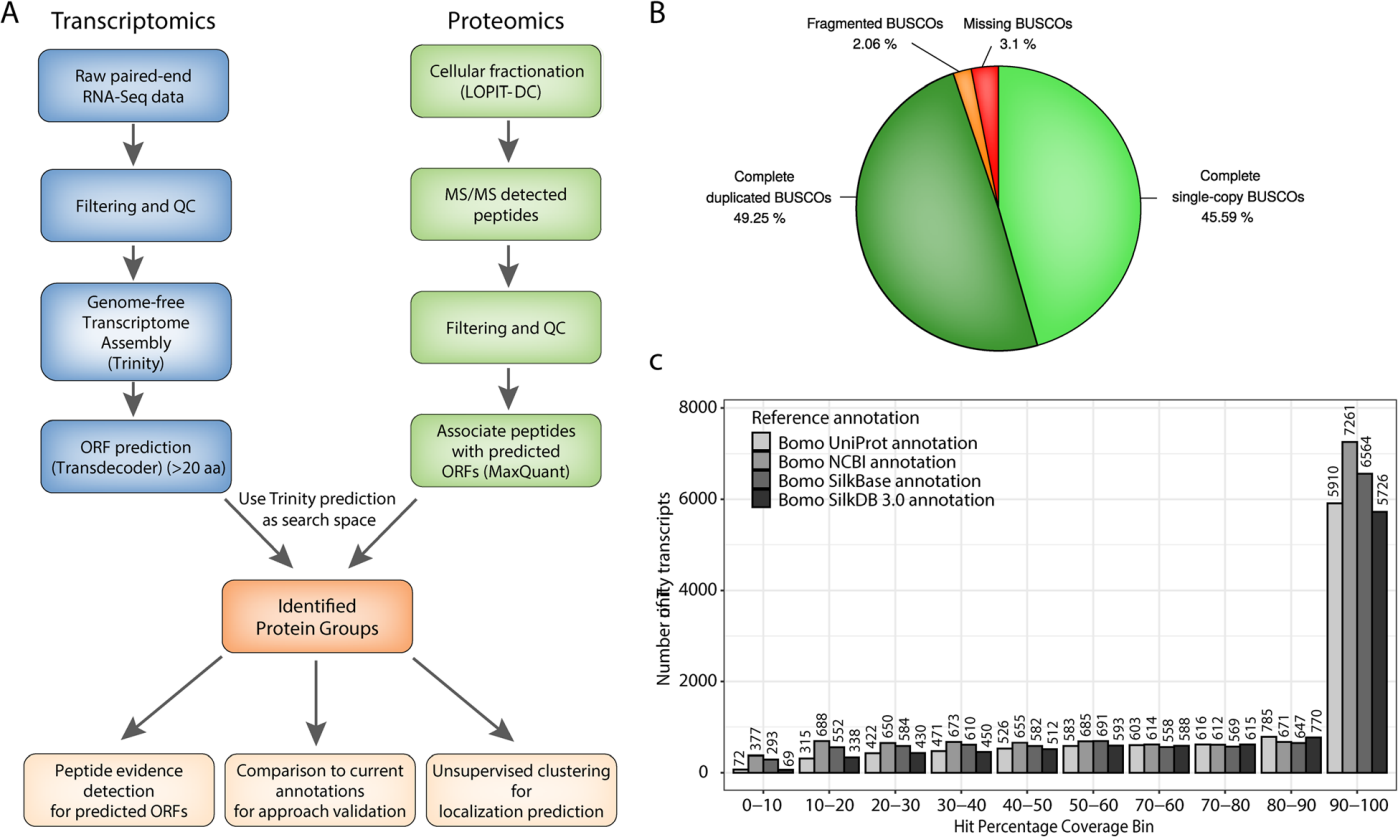
Genome-free transcriptome assembly approach and assessment of annotation quality. **a.** Overview of the proteotranscriptomics annotation approach. **b.** Pie-chart of BUSCO analysis based on the BUSCO arthropoda gene set. **c.** Barplot summarizing the results of a full-length transcript comparison between the genome-free Trinity assembly to currently available annotations from UniProt, NCBI, SilkBase and SilkDB 3.0

We generated 167.8 million paired reads (86.2 million reads from poly-A enriched and 81.6 million from rRNA depleted total RNA samples) by Illumina paired-end sequencing of RNA prepared from the BmN4 cell line of *Bombyx mori.* To prove our concept of being able to produce a proper annotation without a genome and due to the unclear genetic background of the cell line, we applied a genome-free *de-novo* approach using the Trinity suite [12]⁠ (Fig. 1a). After quality filtering, adapter trimming and erroneous k-mer removal almost 165 M paired reads and 158,589,380 bases were assembled into 186,401 ‘Trinity transcripts’ constituting 120,287 distinct ‘Trinity genes’. The assembled raw transcriptome represents 98.32% of the input reads, which shows that the assembly is highly representative (Additional file 1: Table S1). The traditional N50 statistics describe the minimal transcript length of transcripts that are assembled from at least 50% of the reads. We found the N50 length for our assembly to be 1553 bases. A better representation excludes lowly expressed transcripts as they might exhibit bigger biases. Hence, we investigated the N50 values across different expression level bins (ExN50) (Additional file 1: Fig. S1). We found that the ExN50 peaks between the 80 and 90 expression percentiles. Thus a better representation is the E90N50 statistic, which represents the minimal transcript length of transcripts in the 90th expression percentile that are assembled from at least 50% of the reads mapping to these transcripts. The E90N50 transcript contig length is 2270 bases (Additional file 1: Table S2). We used TransRate [13]⁠ to validate the quality of the raw assembly (Additional file 1: Table S3). TransRate assesses the accuracy and completeness of a transcriptome assembly using only the input reads. It proceeds by mapping the reads to the assembled contigs, analyzing the alignments, calculating metrics for each individual contig, integrating these contig-level metrics to provide a contig score, and then combining the accuracy of the assembly with the score of each contig to produce an overall assembly score. The crude overall and optimal Transrate assembly score is 0.31 and 0.41, respectively, of which both are in the 70th percentile range of Transrate assembly scores of 155 published *de-novo* assembled transcriptomes [13]⁠ (Additional file 1: Fig. S2). The expression-level-weighted assembly score, which weights each constituent contig score by the relative abundance level of each contig raises to 0.54 validating the high quality of the assembly and indicating that most of the low TransRate scores stem from contigs that are of relatively low abundance.

To extract all potential protein-coding transcripts from the assembled contigs, we applied TransDecoder [14]⁠ and kept transcripts that comprise an open reading frame of at least 20 amino acids. This filtering resulted in a list of 317,031 potential protein-coding open reading frames based on 95,817 individual genes. These potential protein-coding sequences are the basis of our further analysis. Using BUSCO [15]⁠, we detected that our assembled protein coding transcripts cover 94.8% of the arthropod BUSCO gene set (Fig. 1b and Additional file 1: Table S4). Currently, three main initiatives have provided *Bombyx mori* genome assembly and annotation. For precision estimation, we compared our data to the currently available annotations of the different *Bombyx mori* varieties from UniProt UP000005204 with 14,776 gene models [16]⁠, NCBI Annotation Release 101 with 14,998 gene models, SilkBase 2017 with 16,880 gene models [4]⁠ and SilkDB 3.0 with 16,069 gene models [17]. In general, correspondence between current annotations and *de-novo* predicted proteins is high, with the majority of transcripts sharing protein full sequence coverage (90–100%) to the respective UniProt, NCBI, SilkBase and SilkDB 3.0 proteins (Fig. 1c). When analyzing the genetic sequence variation between the BmN4 cell line and the transcripts from the NCBI and SilkBase annotation by mapping the RNA-seq data to the respective CDS sequences of predicted gene models, we found on average an exchange of 1 in 129 bases for NCBI annotations (i.e. 126,300 changes in 16,393,027 bases) and 1 in 105 bases for SilkBase annotations (i.e. 187,534 changes in 19,826,985 bases). The results of both comparisons are highly consistent. Approximately 75% of the detected changes are silent (synonymous) mutations while around 25% have missense (non-synonymous) and 0.4% nonsense effects (Additional file 1: Table S5). These results unveil an unexpected quite large variation between *Bombyx mori* strains and emphasize the importance of applying a genome-free approach to provide exact CDS sequences especially for molecular biology applications.

Functional annotation of TransDecoder predicted protein sequences was performed using Trinotate [18]⁠ including blastp searches against all model species Swissprot databases, HMMER to identify protein domains, signalP to predict signal peptides, tmHMM to predict transmembrane regions and RNAMMER to identify rRNA transcripts. Furthermore, we used deeploc to predict protein localizations from the respective protein sequences. All functional annotations are included in the annotation file (Additional file 2: Table S10).

### High resolution mass spectrometry data provides peptide evidence for protein coding transcripts

**Fig. 2:**
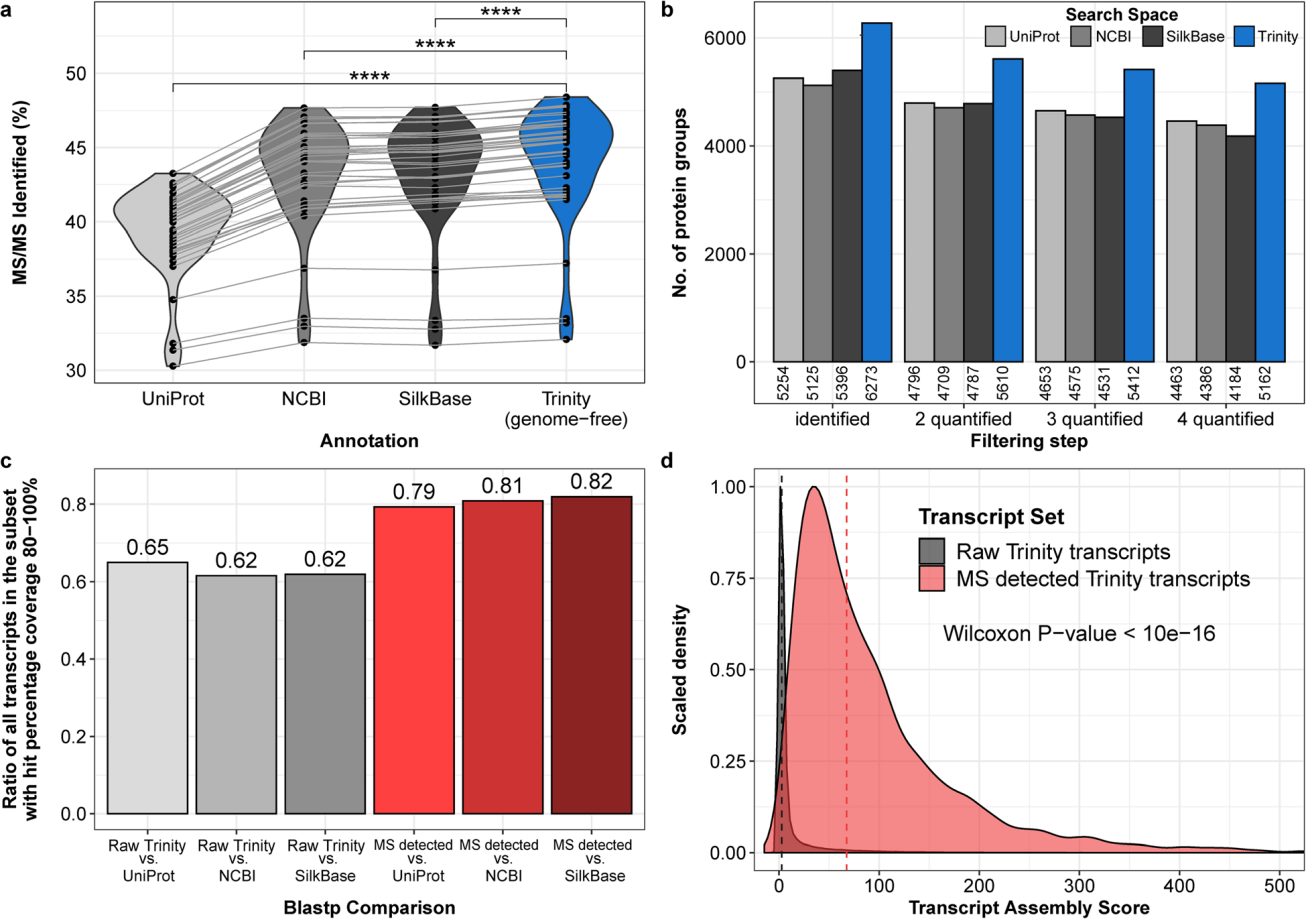
High resolution mass spectrometry provides evidence for superior genome-free annotation. **a.** Violin plots show distribution of identified MS/MS spectra (in percent) for each database used. With identical raw proteomic data the genome-free Trinity annotation shows significantly higher identified tandem MS spectra percentages than the four currently available annotations from UniProt, NCBI, SilkBase and SilkDB 3.0. Grey lines connect percentages stemming from the same MS run. **** indicates two-sided paired Wilcoxon signed rank test *p*-values below 0.0001. **b.** Barplot showing number of protein groups identified after different filtering steps with UniProt, NCBI, SilkBase, SilkDB 3.0 and genome-free Trinity annotation. The Trinity annotation shows higher numbers of identified protein groups for identification and quantification (including replicates). **b.** Barplot of the ratio of transcripts with a hit percentage coverage of more than 80% when compared to current *Bombyx mori* annotations. Grey bars include all Trinity annotated transcripts and red bars represent transcripts that have peptide evidences detected by MS. **d.** Scaled density plot showing distribution of transcript assembly scores of all Trinity annotated transcripts (gray) and transcripts with peptide evidences detected by MS (red). Dashed vertical lines indicate the median assembly score of each subset

In order to provide evidence for the protein coding capability of our predicted protein coding open reading frames (ORF), we performed mass spectrometry measurements of protein extracts from the BmN4 cell line. Using LOPIT-DC [11]⁠ as a strategy to fractionate our sample, we aimed to increase protein detection depth, while also gaining cellular localization information for the detected protein sequences. Our full *Bombyx mori* proteome data set with 10 fractions contained 3,685,257 MS/MS spectra. Using the predicted open reading frames of the Trinity transcriptome assembly as search space for the mass spectrometry data, we noted a higher rate of identified MS/MS spectra compared to using the four currently available protein annotations from UniProt, NCBI, SilkBase and SilkDB 3.0 (two-sided paired Wilcoxon signed rank test, *p* = 3.7*10^− 8^,*p* = 4*10^− 8^, p = 3.7*10^− 8^ and *p* = 3.71*10^− 8^, respectively (Fig. 2a). Applying stringent filtering criteria to have at least 2 identified peptides (at least one of them being unique), we identified a total of 6273 protein groups (fasta files of the CDS and protein sequences of these proteins are provided Material S1 and S2, respectively). This was 16, 18, 14 and 24% higher than for the currently available UniProt (5254 identified protein groups), NCBI (5125 identified protein groups), SilkBase (5396 identified protein groups) and SilkDB 3.0 annotations (4794 identified protein groups) (Fig. 2b), emphasizing the power of strain specific gene sequences to increase proteome coverage and also validating the high quality of our genome-free *de-novo* transcriptome assembly. In order to investigate whether peptide identification could be hindered by provided protein annotations that include strain specific differences in protein sequences, we extracted peptide identification for relevant proteins from both Trinity and the most similar SilkBase annotations and chose as representatives those pairs that have an overlap of more than 80% in sequence but are not 100% identical in their protein sequence. We extracted all missense mutations that were identified for these annotation pairs and calculated for the respective locations the proportion of peptides that were not identified in the SilkBase search, although they could be identified in our de-novo Trinity annotation. We found that 88% of peptides assigned to predicted missense mutations (2325 out of 2653 peptides in 1988 protein groups affected) indeed hamper peptide identification when using the SilkBase annotation as search base.

Detected protein groups show improved quality statistics when compared to the raw in silico predicted potential protein coding transcripts, e.g. better overall correspondence with current UniProt, NCBI, SilkBase and SilkDB 3.0 annotations (Fig. 2c, Additional file 1: Fig. S3A), higher assembly scores (Fig. 2d) and longer transcript lengths (Additional file 1: Fig. S3B). We further observed that assembled contigs with high TransRate scores are indeed enriched with identification by mass spectrometry emphasizing the validity of the scoring approach used by TransRate, which is based on the raw read alignment features only (Additional file 1: Fig. S3C).

Although the correspondence between our annotation and the annotation provided by NCBI and SilkBase is overall high (Fig. 2c), there are still almost 20% of predicted coding sequences that correspond with less than 80% hit percentage coverage to the current annotations. We noted that some of the current SilkBase annotated transcripts are split into several (mostly two) separated genes in our annotation. This observation can have two main explanations: either the current gene annotations are interdependent (SilkBase includes NCBI annotations in the prediction process) and thus an erroneous annotation from earlier predictions could have been transferred to the newest SilkBase annotation, or, the separation of the non-corresponding annotations in our genome-free approach is wrong. To decide between these two possibilities, we checked the RNA-seq read coverage across the gap between two separated proteins in our annotations that were suggested by SilkBase to be a single protein (941 pairs in total corresponding to 631 SilkBase genes). For this we investigated the RNA-seq reads coverage for each of the relevant pairs of our Trinity predicted proteins and the respective genomic gap between these using the SilkBase genome assembly. Overall we observe that 76% of the Trinity annotated splits (629 out of 826 protein pairs) are well supported by clear gaps in the RNA-seq raw data alignment at the split site (Fig. S4 A and B). Only 5.5% of all Trinity predicted proteins (348 unique proteins in 195 protein pairs out of 6273 detected proteins) do not show an evident gap in read coverage and hence likely have been falsely split in the annotation process (Additional file 1: Table S6). Our set of identified ORFs also includes 188 predictions that have a less than 85% hit coverage with a SilkBase annotation entry. The length differences are shown in Additional file 1: Fig. S5A. In order to investigate if the shorter ORFs are supported by read mapping data, we calculated the difference between the read mapping frequency in the ORF region with the coverage at the edges of the transcripts. 124 (66% of the short ORFs) identified proteins show an evident absence of reads at the edges of the transcripts, while the remaining 64 might have been falsely split as we could observe mapped reads adjacent to the edges (Additional file 1: Fig. S4C). Based on these observations, we conclude that our method has a precision rate of at least 93.4% (5861 out of 6273) for assigning individual genes. The respective categorizations into “high correspondence”, “evidently split”, “probable false split”, “evidently shorter than SilkBase” and “probably falsely shorter than SilkBase” have been included in the annotation table for clarity (Additional file 2: Table S10). Furthermore, we found mass spectrometry evidence for 164 predicted proteins that have been missed in any of the current annotations (marked with “newly annotated” in the annotation Additional file 2: Table S10, peptide evidence information for all novel proteins are provided in Additional file 4: Table S12). Another group of genes (513 genes (8% of all ORFs detected by MS); marked with “longer than SilkBase”) are longer than annotated in the current SilkBase annotation, however differences are mostly neglectable (Additional file 1: Fig. S5B). We provide a website (http://butterlab.org/bombyxviewer) which incorporates data regarding all ORFs with mass spectrometric evidence including transcript and protein sequences and a genome viewer based on the SilkBase genome showing gene structure and individual RNA-seq read mapping. Peptide evidence information and annotated MS-MS spectra of newly identified proteins are therewith also provided for download.

### Subcellular localization of proteins determined by LOPIT-DC

**Fig. 3:**
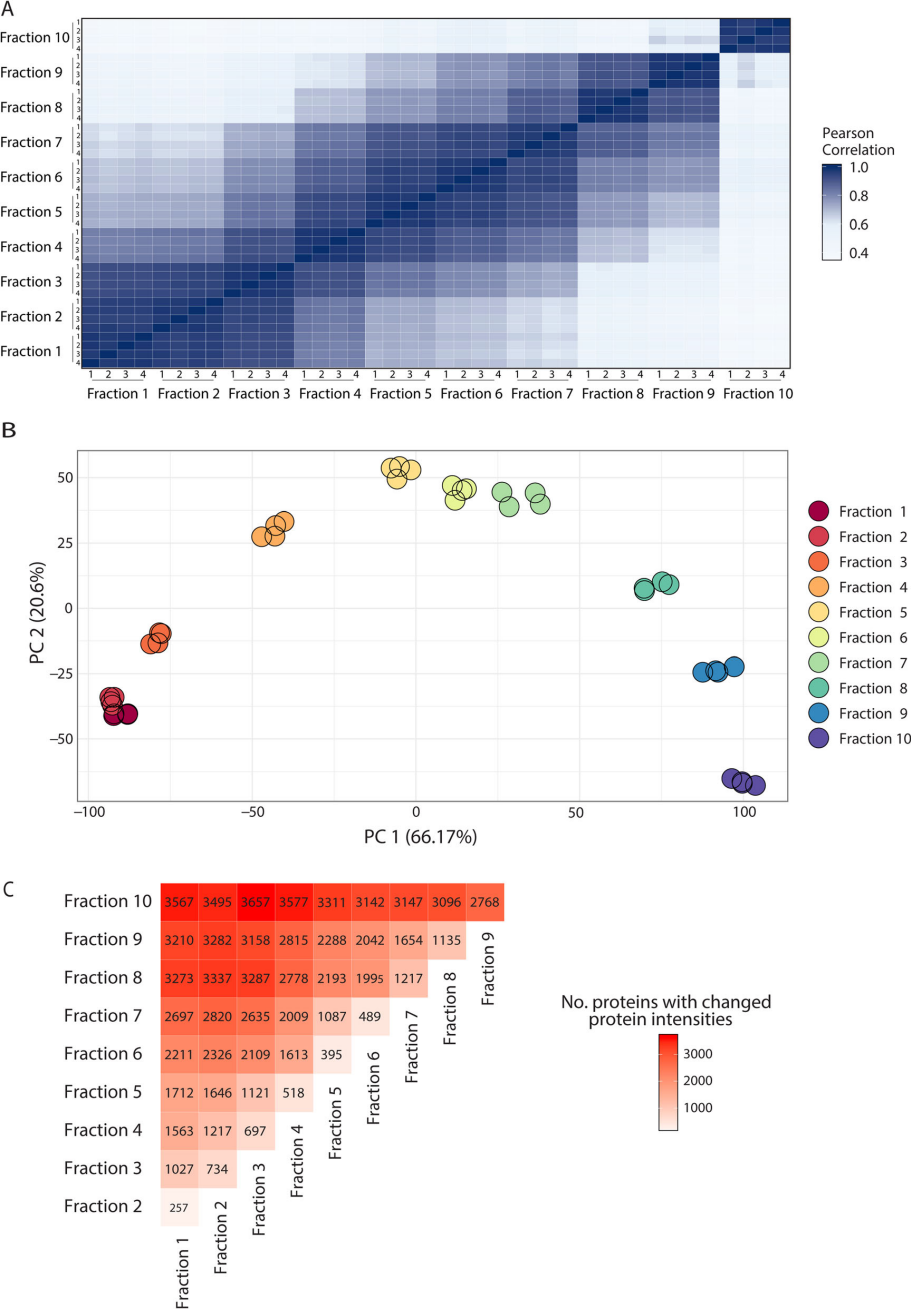
Spatial proteomics unveils subcellular localization of *Bombyx mori* proteins. **a.** Heatmap of all pairwise comparisons (pearsons correlations) shows high concordance between replicates and relatedness of adjacent fractions. **b.** Principal component plot based on the 1000 most dynamic protein groups demonstrates differences of fractions and similarity of replicates. Replicates (same color) cluster together and (except for fraction 1 and 2) farther away from the other fractions. **c.** Summary heatmap of number of proteins with significantly different protein intensities across fractions

To assign the *Bombyx mori* proteins to sub-cellular compartments, we performed label-free quantitative spatial proteomics adapting the recently released LOPIT-DC protocol [11]. Using differential centrifugation steps, we generated 10 subcellular fractions in independent quadruplicates. Fractionation replicates correlate very well (average Pearson correlation coefficient > 0.97) (Fig. 3a) and cluster together in the first two PCA dimensions (Fig. 3b). Within the gradient, fraction series [1–9] are similar to each other, while fraction 10 is most different from all others. This is consistent with fraction 10 constituting an acetone precipitation of all proteins that were not separated in the previous fractionation steps. Analyzing significant changes in pairwise comparisons of all fractions, efficient separation can be recapitulated, i.e. an increasing diversity of proteins can be observed throughout subsequent fractionation steps (Fig. 3c). LFQ data and differential expression statistics across fractionation samples can be found in Additional file 3: Table S11.

**Fig. 4:**
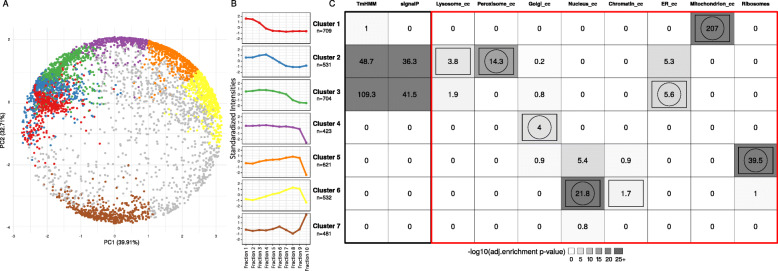
Unsupervised clustering of fractionation mass spectrometry data. **a.** Principal component plot of components 1 and 2 calculated from standardized average protein intensities. Standardized protein intensities across fractionation samples were clustered using SOM (self-organizing maps). Proteins in PCA space are colored according to their assignment to one of eight distinct clusters (see color code in b). 795 proteins could not be associated with any of the clusters (gray colored dots). **b.** Line plots of the median standardized protein intensities across fractionation steps of the different clusters assigned by SOM (see a). **c.** Heatmap summarizing enrichment analyses of cellular localization annotations in the respective clusters. Color darkness corresponds to levels of enrichment (−log10 of adjusted *P*-values). Rectangles and circles indicate highest enrichment for the corresponding localization and gene clusters, respectively. General and more specific localization categories are framed in black and red, respectively

We applied an unsupervised clustering approach to detect unique fractionation profiles. Self-organizing map (SOM) clustering of the normalized protein intensities of all proteins showing significant changes in at least one of the pairwise fractionation comparisons and appropriate clustering assessment and filtering (see Methods and Additional file 1: Fig. S6) revealed 8 main clusters encompassing 3942 protein groups as depicted in Fig. 4a. The mean expression profiles across fractionation of the different clusters are depicted in Fig. 4b (and in Additional file 1: Fig. S7 for individual genes). In order to characterize the different clusters in terms of their potential subcellular localization or function, enrichment of specific categories as determined by signalP (prediction of signal peptides), tmHMM (prediction of transmembrane regions) and the Gene Ontology cellular-component (GO_cc) annotation were calculated (Fig. 4c, Additional file 1: Table S7). Clusters 1–4 represent proteins that show relatively high intensities in the early fractionation steps with diminished intensities in later steps. Generally, membrane associated proteins (framed with black box in Fig. 4c) are highly enriched in clusters 2 and 3. To get a more specific insight into the subcellular localization, we subsequently checked for enrichment of the GO_cc terms ‘lysosome’, ‘peroxisome’, ‘golgi apparatus’, ‘nucleus’, ‘chromatin’, ‘endoplasmic reticulum’ (ER), ‘mitochondrion’ and ‘ribosomes’, which were inferred by orthology to well-annotated model organisms in each cluster (framed with red box in Fig. 4c). The most prominent enrichment was observed for cluster 1, which exhibits high levels in the first three fractions and low levels in later fractions. This cluster is highly enriched with mitochondrial genes (Fisher’s exact test, *P* = 10^− 197^, fold-enrichment = 35.56). Cluster 2 shows reduced intensity after fractionation step 4 and is enriched with peroxisome, ER and lysosome annotated proteins (Fisher’s exact test, *P* = 10^− 16^, *P* = 10^− 6^ and 10^− 5^, fold-enrichment = 24.14, 3.2 and 4, respectively). Cluster 3 has lower intensity starting at fractionation step 5 and represents mainly endosplasmatic reticulum (ER) proteins (Fisher’s exact test, *P* = 10^− 7^, fold-enrichment = 3.37). The profile of cluster 4 shows reduction of protein intensities after fractionation step 8 and contains proteins from Golgi apparatus (Fisher’s exact test, *P* = 10^− 5^, fold-enrichment = 2.9). The second highest enrichment could be observed for cluster 5, where measured protein intensities peak in fraction 7–9 and which is highly enriched with ribosomal 40s and 60s proteins (Fisher’s exact test, *P* = 10^− 25^, fold-enrichment = 11.76). Cluster 6 encompasses proteins with low abundance in the initial fractionation steps, which increase until step 9 and decline to minimal levels in fraction 10. This cluster exhibits a mixed enrichment profile of nuclear and chromatin associated proteins, but also with ribosomal proteins (Fisher’s exact test, *P* = 10^− 14^, *P* = 10^− 3^ and *P* = 10^− 13^, fold-enrichment = 2.62, 2.97 and 6.92, respectively). Cluster 7 is the only cluster, that show exclusively high enrichment with nucleus associated proteins (Fisher’s exact test, *P* = 10^− 19^, fold-enrichment = 3.18). Cluster 8, which shows abrupt protein intensity increase in the last two fractions couldn’t be associated with any of the known localizations and hence was not further analyzed. Overall the cellular localization profiles of the different clusters correspond very well (average Spearman’s correlation coefficient = 0.88) with those established in the LOPIT-DC method paper [11] where the assignment was established using experimentally validated marker genes (Additional file 1: Fig. S8). Further, orthologous proteins of established *Drosophila melanogaster* cellular compartment marker genes show highly coherent profiles to the ones established by our unsupervised approach (Additional file 1: Fig. S9). This shows that the fractionation strategy is robust and widely applicable. Comparing the fractionation profile of each protein to each of the clusters enables localization prediction also for proteins that could not be annotated properly by previous in silico analysis. The resulting clusters allow to assign proteins especially to the following 6 compartments (in descending order of certainty): Mitochondria (cluster 1), Ribosome (cluster 5), Nucleus (cluster 7), Peroxisome (cluster 2), Endoplasmic reticulum (cluster 3) and Golgi apparatus (cluster 4). For each individual MS detected protein, we calculated the correlations between its expression profile across fractionations and the predicted median profile of the above depicted localization. These correlation values are provided as confidence score for the localization probability of each protein. The information for each detected protein, including all relevant information such as transcripts type, length, score, annotations and localization categorization weights are provided in Additional file 2: Table S10). All CDS and protein sequences of the assembled and identified proteins are provided in Additional files 5 and 6.

## Discussion

We here show that by combining comprehensive RNA-seq data and high-resolution mass spectrometry data, we achieve a comparable and even slightly better annotation of protein-coding genes in *Bombyx mori* than previous efforts based on genome or transcriptome guided *de-novo* strategies. Even in the comparison of our genome-free to our own genome-guided assembly using the same raw RNA-seq and mass spectrometry data a slightly better performance can be observed in the genome-free approach (see Additional file 1: Table S9). This fact emphasizes the importance of using genome-free approaches in conditions were provided genomes are suspected to stem from different genetic backgrounds as the measured system.

Our extensive comparison between our genome-free proteotranscriptomics annotation and the provided annotations from UniProt, NCBI, SilkBase and SilkDB 3.0 showed that UniProt and SilkDB 3.0 currently provides the annotation with the weakest performance in BmN4 cells. Gene annotations provided by NCBI and SilkBase are more comprehensive, however still do not report the full protein-coding potential demonstrated by the elevated percentage of identified MS-MS spectra in our tailor-made assembly (Fig. 2a) and the identification of 164 new proteins in this study. As BmN4 cells were derived from the ovary of a female animal, we were curious to know if any of the newly identified proteins might be located on the female-determining *Bombyx mori* chromosome W [19]. Mapping the CDS sequences of the respective proteins to the sequences of the W chromosome did not result in any localization, while they did map rather evenly dispersed to all other chromosomes (see Additional file 1: Table S10). In fact, none of the identified proteins mapped to chromosome W sequences recapitulating the current knowledge that this chromosome is depleted of protein coding genes [20]. Although the correspondence between our annotation and NCBI and SilkBase is overall high, there are still 20% of coding sequences with less than 80% hit length, mostly attributed to split genes in our assembly. Performing a detailed investigation of the gap region, we could provide evidence that in 80% of cases the split version in our assembly is supported, improving gene models for 451 genes.

Many studies have shown that small proteins (≤100 amino acid residues) can be involved in important biological processes, including cell signaling, metabolism, and growth [21]⁠. However, they are underrepresented in many genome annotations as they are notoriously hard to predict because of their small ORF size [21]⁠. To validate these small peptides, we kept all ORF with at least 20 amino acids. Indeed, we detected 308 small proteins (5% of all protein groups identified) with at least two peptides (one of them unique) and many of them reaching relatively high expression levels, however the overall expression levels are lower than for longer proteins (Additional file 1: Fig. S10).

Additionally, we used the recently published LOPIT-DC approach [11]⁠ to provide experimental data for localization of our detected proteins. The high correspondence to the LOPIT-DC results, despite changing from TMT to LFQ and using cell lines from different species (human osteosarcoma U2OS vs. *B. mori* BmN4), indicate a universal applicability of the fractionation protocol and the resulting data. The resulting clusters allow assigning proteins especially to the following compartments: Mitochondria, Ribosome, Nucleus, Peroxisome, Endoplasmic reticulum and Golgi apparatus.

The current fractionation approach allowed us to detect peptide evidence for more than 6200 proteins. If more comprehensive databases are of interest, these limits may be overcome by using more diverse conditions or several different tissues for extraction of transcriptomic and proteomic data. While we here provide annotation for *Bombyx mori*, this approach is readily applicable to any species, including more complex organisms such as vertebrates and plants. While possible parameters might need to be adapted such as even deeper RNA-seq data, the high fraction of non-coding genome regions or highly repetitive structure that make genome assembly challenging can be disregarded.

Our developed proteotranscriptomics approach improves current gene annotations and provides the exact gene sequences for other applications such as gene amplifications via cDNA or planning CRISPR guides around the translation start site.

Importantly, the proposed annotation approach readily works without any genome reference and hence provides a precise, time- and cost-efficient method to construct annotations for protein-coding genes in any species where properly sequenced genomes are still out of reach.

## Conclusions

Combining in-depth transcriptome sequencing and high resolution mass spectrometry, we here use proteotranscriptomics to improve gene annotation of protein-coding genes in the *Bombyx mori* cell line BmN4 which is an increasingly used tool for the analysis of piRNA biogenesis and function*.* Using this approach we provide the exact coding sequence and evidence for more than 6200 genes on the protein level. We show that proteotranscriptomics is an efficient, cost-effective and accurate approach to improve previous annotations or generate new gene models. As this technique is based on de-novo transcriptome assembly, it provides the possibility to study any species also in the absence of genome sequence information.

## Methods

### Experimental design

To build a genome-free proteotranscriptomics-based gene annotation we combined two types of data. First, RNA-seq data of polyadenylated mRNA entities combining poly-A enriched and rRNA depleted samples from *Bombyx mori* BmN4 cell line was used to predict potential protein-coding genes. Secondly, MS/MS spectra data was used to find evidence for the predicted protein-coding genes. Profiling subcellular localization of proteins in *Bombyx mori* cells was performed using the LOPIT-DC strategy [11]⁠. The procedures were performed with four biological replicates based on the high level of reproducibility between replicates (average Pearson correlation coefficient > 0.97). Results are represented as averages of the biological replicates. We used Trinity for genome-free de novo RNA assembly and the MaxQuant data analysis platform [22]⁠ for quantitative proteomics analysis.

### Cell propagation and RNA extraction

The *Bombyx mori* larval ovary-derived cell line BmN4 (RRID:CVCL_Z634) [7]⁠ was kindly provided by the Ketting group (Institute of Molecular Biology, Mainz, Germany)). Cells were cultured in Insect media IPL-40 (Pan Biotech) with 10% heat-inactivated FBS (Sigma) and 1x Penn-Strep (Sigma) at 27 °C.

For RNA-sequencing total RNA was extracted from 10 million cells using the RNAeasy Mini Kit (Qiagen) according to standard protocol. RNA integrity was tested by agarose gel electrophoresis and Bioanalyzer (RNA Nano Assay). RNA was quantified using Qubit.

### RNA-seq measurements

RNA-sequencing libraries were prepared from total RNA using two different RNA enrichment protocols: 1. poly(A) purification using Illumina TruSeq stranded mRNA LT Sample Prep Kit following Illumina’s standard protocol (Part # 15031047 Rev., E). [polyA-enriched] 2. depletion of ribosomal RNA using Illumina TruSeq stranded Total RNA LT Sample Prep Kit following Illumina’s standard protocol (Part # 15031048 Rev. E) [ribo-minus].

The libraries were prepared with a starting amount of 1000 ng and amplified in 10 PCR cycles and profiled using a DNA 1000 Chip on a 2100 Bioanalyzer (Agilent technologies) and quantified using the Qubit dsDNA HS Assay Kit, in a Qubit 2.0 Fluorometer (Life technologies). The two libraries were pooled together in equimolar ratio and sequenced on 1 NextSeq 500 Midoutput FC, PE for 2 × 79 cycles plus 7 cycles for the index read. The measurements of polyA-enriched and ribo-minus RNA resulted in 86,178,436 and 81,597,503 paired-end reads of length 80 bp, respectively. We assayed mycoplasma contamination by aligning all raw RNASeq forward reads to the genomes of *A.laidlawii, M.arginini, M.fermentans, M.hominis, M.hyorinis* and *M.orale*. The maximum percentage of uniquely mapped reads is below 0.00026% and therefore a contamination can be excluded (see Table S8).

### Transcriptome assembly

The two RNA-seq datasets (polyA-enriched and ribo-minus RNA) were used in combination to assemble the transcriptome. First, both raw fastq files were cleaned from erroneous k-mers using Rcorrector [23]⁠ and the specialized scripts from TranscriptomeAssemblyTools (FilterUncorrectablePEfastq.py). Secondly, adapter sequences were removed using TrimGalore (a wrapper around Cutadapt [24]⁠ and FastQC [25]⁠) and reads were filtered to include only pairs consisting of proper pairs of minimum length of 36 nts each. These clean-up steps removed only 2% of the paired reads. The fastq files were then combined. For the genome-guided assembly raw RNA-seq data was mapped to the *Bombyx mori* genome provided by SilkBase (http://silkbase.ab.a.u-tokyo.ac.jp/cgi-bin/index.cgi) using STAR (version 2.5.4b) [26]⁠. The raw RNA-seq or mapped data was used for a genome-free de novo or genome-guided assembly approach using the Trinity suite (Trinity version 2.4.0) [12]⁠ with the following parameter setting: [for genome-free: --seqType fq --SS_lib_type RF --min_kmer_cov 1], [for genome-guided: Trinity –genome_guided_bam –genome_guided_max_intron 30,000 --genome_guided_min_coverage 2]. The resulting Trinity fasta files were then further processed with TransDecoder version 5.4.0 [14]⁠ to predict potential protein coding transcripts using a length threshold of 20 amino acids. The resulting peptide fasta files were used as search space in subsequent steps for mass spectrometry data analysis.

### Quality check of transcriptome assembly

The quality of the assembled transcriptome was assessed using several different state of the art approaches. These included general metrics of number of assembled transcripts, mean, median and Ex90N50 transcript lengths. The alignment rate of the raw reads to the assembly was calculated using Bowtie2 (version 2.3.4.3) [27]⁠ and dedicated scripts provided by Trinity (Trinity version 2.4.0) [12]⁠. BUSCO (version 2.0) [15]⁠ with the arthropodae BUSCO database was used to assess the completeness of the assembly. Transrate scores and additional quality metrics were established using TransRate (version 1.0.3) [13]⁠. Coherence with current annotations was measured using a combination of blastp (BLAST+ version 2.8.1) [28]⁠ and Trinity tools (Trinity version 2.4.0) [12]⁠. For RNA-seq coverage validations the combined cleaned RNA-seq data was mapped to the SilkBase genome assembly using STAR (version 2.5.4b) [26]⁠. Coverage per base was calculated using bedtools (version 2.26.0) [29]⁠ using the -pc option to also account for intronic alignment. Then using customized R (version 3.5.3) [30]⁠ scripts the average coverage per transcript or gap region was extracted (Fig. S4).

### Annotation of identified transcripts

Functional and domain annotations were produced using Trinotate (version 3.1.1) [18]⁠ combining the following applications: HMMER (version 3.2.1) [31]⁠ to identify protein domains, signalP (version 5.0) [32]⁠ to predict signal peptides, TMHMM (version 2.0c) [33]⁠ to predict transmembrane regions, RNAMMER (version1.2) [34]⁠ to identify rRNA transcripts in addition to infer Gene Ontology and KEGG terms from orthologies established by BLAST+ (version 2.8.1) [28]⁠ with a swissprot database of all major model species. Further, localization predictions from protein sequences of the assembly were calculated using deeploc (version 1.0) [35]⁠.

### Detection of variation level between NCBI and SilkBase protein coding sequences and BmN4 RNASeq data

In order to characterize the level of variation between the *Bombyx mori* coding sequences in the NCBI annotation and the BmN4 specific transcriptome, we first mapped the RNA-seq data also used for transcriptome assembly to NCBI coding sequences using STAR aligner (version 2.5.4b) [26]⁠. We then used GenomeAnalysisTK (version 3.8–0-ge9d806836) [36]⁠ to extract sequence variation information into a vcf file. SnpEff (version 4.4) [37]⁠ was used to annotate all sequence changes regarding their type (single nucleotide polymorphism (snp), deletion, insertion), their impact (low, moderate, high severeness) and their functional class (missense, nonsense, silent mutation). Results of this analysis are summarized in Table S5.

### Subcellular fractionation

Subcelluar fractionation of *Bombyx mori* cells was performed in quadruplicates and based on the LOPIT-DC strategy [11]⁠ with some modifications. Per replicate 70 million BmN4 cells were collected and suspended in 4 ml lysis buffer (0.25 M sucrose, 10 mM HEPES pH 7.5, 2 mM EDTA, 2 mM Magnesium acetate, Roche complete protease inhibitors). Cells were dounced in a 7 ml glass douncer with 50 strokes using a type B pestle. Samples were fractionated using centrifugation speed and times as indicated in the table below following the LOPIT-DC strategy [11]⁠. For centrifugation steps a Heraeus Fresco 21 centrifuge (Thermo) or a Optima Max-XP benchtop centrifuge (Beckmann) with a TLA 100.3 rotor were used. Supernatant of the 9th fractionation step was precipitated using ice cold acetone and the pellet was resuspended in 50 mM HEPES/KOH pH 7.9 (represents fraction 10).

### Mass spectrometric sample preparation and measurement

50 μg protein from each fraction were loaded on a 4–12% NOVEX Bis-Tris PAGE gel (Thermo) and separated for 7 min at 180 V in 1x MES buffer (Thermo). Proteins were fixated and stained with Coomassie. After destaining with water, in-gel digest preparation and MS stage tip purification were performed as previously described [38]⁠, [39]⁠. Peptides were analyzed by nanoflow liquid chromatography on an EASY-nLC 1000 system (Thermo) coupled to a Q Exactive Plus mass spectrometer (Thermo). Peptides were separated on a C18-reversed-phase column packed with Reprosil aq1.9 (Dr. Maisch GmbH), directly mounted on the electrospray ion source of the mass spectrometer. We used a ca. 200 min gradient from 2 to 60% acetonitrile in 0.1% formic acid at a flow of 225 nL/min. The Q Exactive Plus was operated with a Top10 MS/MS spectra acquisition method per MS full scan.

### Protein identification and label free quantification of protein intensities

MaxQuant (version 1.5.2.8) [22] was used for raw file peak extraction and protein identification against the following databases individually: UniProt UP000005204 (14,776 entries) [16]⁠, NCBI *Bombyx mori* Annotation Release 101 (14,998 entries), SilkBase 2017 (16,880 entries) [4]⁠, SilkDb 3.0 (16,069 entries) [17] or our Trinity-based ORF library (317,031 entries). Protein quantification was performed with MaxQuant using the label free quantification (LFQ) algorithm [40]⁠. The following parameters were applied: trypsin as cleaving enzyme; minimum peptide length of seven amino acids; maximal two missed cleavages; carbamidomethylation of cysteine as a fixed modification; N-terminal protein acetylation and oxidation of methionine as variable modifications. Peptide mass tolerance was set to 20 ppm and 7 ppm was used as MS/MS tolerance. Further settings were: “label-free quantification” with “FastLFQ” disabled, “match between runs” with time window 0.7 min for matching and 20 min for alignment; peptide and protein false discovery rates (FDR) were set to 0.01; common contaminants (standard MaxQuant contaminant list including trypsin, keratin etc.) were excluded. Detailed settings are available in the respective parameter files (uploaded to ProteomeXchange (www.ProteomExchange.org) via the PRIDE [41]⁠ partner repository with the dataset identifier PXD014626). MaxQuant LFQ data was further processed using in-house developed tools based on R (version 3.5.3) [30]⁠. This included filtering out marked contaminants, reverse entries and proteins only identified by site. Protein groups with no unique and less than two peptides were removed. Protein group averages were calculated from proteotypic peptides. Prior to imputation of missing LFQ values with a beta distribution ranging from 0.1 to 0.2 percentile within each sample, the values were log2 transformed. For protein groups consisting of more than one Trinity annotation, we chose the longest as representative of the group for further analysis.

### LFQ data analysis and unsupervised clustering

For overall statistical quality calculations, we calculated proportions of assembly scores, transcript lengths and correspondences with UniProt, NCBI, SilkBase and SilkDB 3.0 *Bombyx mori* annotations. For clustering purposes, we focused only on protein groups that have four measured LFQ levels in at least one of the fractions and showed significant enrichment in at least one of the pairwise fraction comparisons ending up in 5058 out of 5610 protein groups (90%). The mean LFQ data of the four biological replicates data was standardized protein-group-wise by adding the mean of all average fraction intensities and dividing by the standard deviation between all average fraction intensities of the respective protein group. An unsupervised machine learning approach was used to cluster all standardized profiles. We applied the kohonen R package [42]⁠ to build a self-organizing-map (SOM), i.e. to build an artificial neural network that is trained using unsupervised learning to produce a two-dimensional, approximated grouping of the input profiles. The standardized data was initially assigned to a SOM matrix of 12 basic clusters. After combining four clusters with high intra-cluster differences (mean differences above the 75%-ile intra-cluster distances of all protein groups; see Additional file 1: Fig. S6) into one cluster with uncategorized profiles, eight distinct clusters representing similar profiles remained. These clusters were ordered according to euclidean distances in the first two-component PCA space using the TSP (travelling salesman problem) R package (version 1.1–7) [43]⁠. Using localization data retrieved through orthology and sequence screening approaches as described earlier, we checked for enrichment of categories relevant for cellular localization, namely TmHH (sequence based transmembrane region predictions), signalP (sequence based signal peptides identification), Gene Ontology associations based on blast orthology associations for cellular component annotations lysosome, peroxisome, golgi, nucleus, chromatin, endoplasmic reticulum (ER), mitochondrion and ribosome (based on 40S and 60S ribosomal proteins) annotations. Enrichment scores of each category and each fractionation profile cluster were calculated using Fisher’s Exact test (one-sided test, alternate-hypothesis: cluster genes contain more genes belonging to the tested category than non-cluster genes). Correlation of the identified cluster profiles were compared to the profiles of the same cellular localization categories from the original LOPIT-DC paper [11] by calculating the spearman correlation between the median standardized LFQ profiles of our data and the standardized median TMT profiles of the TMT data from human osteosarcoma U-2 OS cell line across fractionations (normalized TMT data is provided in Supplementary data 1 of previous publication [11]⁠). Respective profiles and correlations are shown in Additional file 1: Fig. S8. *Drosophila melanogaster* cellular compartment marker genes were downloaded from the pRoloc bioconductor package [44] and blasted against our denovo protein database using blastp (BLAST+ version 2.8.1) [28] to find the respective orthologous genes. Newly identified genes were mapped to SilkDB 3.0 genome assembly and sequences of the W chromosomes using gmap version 2019-03-04 [45]. W chromosome sequences were downloaded from GenBank nucleotide database accession numbers AB251908–AB251914.

## Supplementary information

**Additional file 1 Supplemental Fig. S1**. Distribution of length distributions across RNA expression level bins. **Supplemental Fig. S2**. Comparison of Transrate assembly scores to other publicly available assemblies. **Supplemental Fig. S3**. Overall MS detected transcripts show improved assembly features. **Supplemental Fig. S4**. RNA-Seq coverage assay for the detection of falsely split genes. **Supplemental Fig. S5**. Densityplot of deviations from SilkBase annotation for longer annotated genes. **Supplemental Fig. S6**. Intracluster distances of SOM clusters can be used to filter out clusters that have high variability within the cluster. **Supplemental Fig. S7**. Depiction of expression profiles of all genes separated into respective clusters. Supplemental Fig. S8: Comparison of fractionation profiles of the respective cellular compartments in original LOPIT-DC TMT data and our Proteotranscriptomics LC-MS-MS approach. **Supplemental Fig. S9**. Comparison of fractionation profiles of orthologs of established *Drosophila melanogaster* cellular compartment markers to the mean dynamics determined by SOM clustering and enrichment analysis. **Supplemental Fig. S10**. Comparison of LFQ expression levels of detected protein groups that are shorter (small proteins) or longer than 20 amino acids. **Supplemental Table S1**. Read representation statistics of the Trinity assembly. **Supplemental Table S2**. Expression bins and transcript lenghts. **Supplemental Table S3**. TransRate analysis results. **Supplemental Table S4**. BUSCO analysis results. **Supplemental Table S5**. *Bombyx mori* NCBI and SilkBase based BmN4 variome. **Supplemental Table S6**. Statistics of correspondence and differences between genome-free and SilkBase annotations. **Supplemental Table S7**. Table including enrichment values for all clusters and all cellular localization categories. **Supplemental Table S8**. Mycoplasma contamination assay. **Supplemental Table S9**. Comparison of genome-free and genome-guided assembly. **Supplemental Table S10**. Mapping statistics of newly identified protein CDS sequences to the sequences of the female-determining chromosome W

**Additional file 2 Table S10**. Functional annotation and characteristics of ORFs

**Additional file 3 Table S11**. Label-free quantification data.

**Additional file 4 Table S12**. Novel protein evidence data.

**Additional file 5.** BOMOPTA annotation CDS sequences.

**Additional file 6.** BOMOPTA annotation protein sequences.

## Data Availability

RNA-sequencing raw data of polyadenylated mRNA entities has been submitted to the Sequence Read Archive (SRA) under SRA identifier SRR9685281. The mass spectrometric proteomics data including all raw and processed data files and MS/MS spectra files has been deposited to the ProteomeXchange Consortium via the PRIDE partner repository [41] with the dataset identifier PXD014626. Genome sequences of the mycoplasma species *A. laidlawii* (Acholeplasma_laidlawii_pg_8a.ASM1878v1.dna.toplevel), *M. arginini* (Mycoplasma_arginini_7264.version_1.0.dna.toplevel), *M. fermentans* (Mycoplasma_fermentans_pg18.ASM20973v1.dna.toplevel), *M. hominis* (Mycoplasma_hominis_atcc_23114.ASM8586v1.dna.toplevel) and *M. hyorinis* (Mycoplasma_hyorhinis_sk76.ASM31363v1.dna.toplevel) were downloaded from Ensemble ftp site (ftp.ensemblgenomes.org). The genome sequence of *M. orale* (GCF_000420105.1_ASM42010v1_genomic) was downloaded from the NCBI ftp site (ftp.ncbi.nlm.nih.gov). The UniProt proteome of *Bombyx mori* was downloaded from UniProt database with accession number UP000005204. The NCBI genome of *Bombyx mori* was downloaded from NCBI database using assembly ID ASM15162v1, the NCBI proteome was downloaded using Annotation Release ID 101. The SilkBase genome assembly (Nov.2016) and protein models (Jan.2017) was downloaded from http://silkbase.ab.a.u-tokyo.ac.jp/cgi-bin/download.cgi. The SilkDB 3.0 genome assembly (chromosome.fa.tar.gz) and protein sequences (protein.fa.tar.gz) were downloaded from https://silkdb.bioinfotoolkits.net/base/download/-1. W chromosome sequences were downloaded from GenBank nucleotide database using accession numbers AB251908–AB251914. All CDS and protein sequences of the assembled and identified proteins are provided in Additional files 5 and 6. The complete Trinity-based ORF database can be downloaded from http://butterlab.org/bombyxviewer.

## Abbreviations

BmN4: *Bombyx mori* N4 cell line
piRNA: Piwi-interacting RNA
RNA-seq: RNA-sequencing
CRISPR: Clustered regularly interspaced short palindromic repeats
LOPIT-DC: Localisation of organelle proteins by isotope tagging after differential ultracentrifugation
rRNA: Ribosomal RNA
BUSCO: Benchmarking Universal Single-Copy Orthologs
CDS: Coding sequences
cDNA: Complementary DNA
ORF: Open reading frame
MS/MS: Tandem mass spectrometry
PCA: Principal component analysis
SOM: Self-organizing maps
GO: Gene Ontology
GO_cc: Gene Ontology cellular compartment
ER: Endoplasmic reticulum
TMT: Tandem mass tag
LFQ: Label-free quantification
TSP: Traveling salesman problem
