# Correction to: Proteotranscriptomics assisted gene annotation and spatial proteomics of *Bombyx mori* BmN4 cell line

**DOI:** 10.1186/s12864-020-07211-8

**Published:** 2020-11-12

**Authors:** Michal Levin, Marion Scheibe, Falk Butter

**Affiliations:** Institute of Molecular Biology (IMB), Ackermannweg 4, 55128 Mainz, Germany

**Correction to: BMC Genomics 21, 690 (2020)**

**https://doi.org/10.1186/s12864-020-07088-7**

Following publication of the original article [1], it was reported that there were errors in Figs. 1 and 2. The correct figures are included in this Correction article and the original article has been updated.

**Fig. 1:**
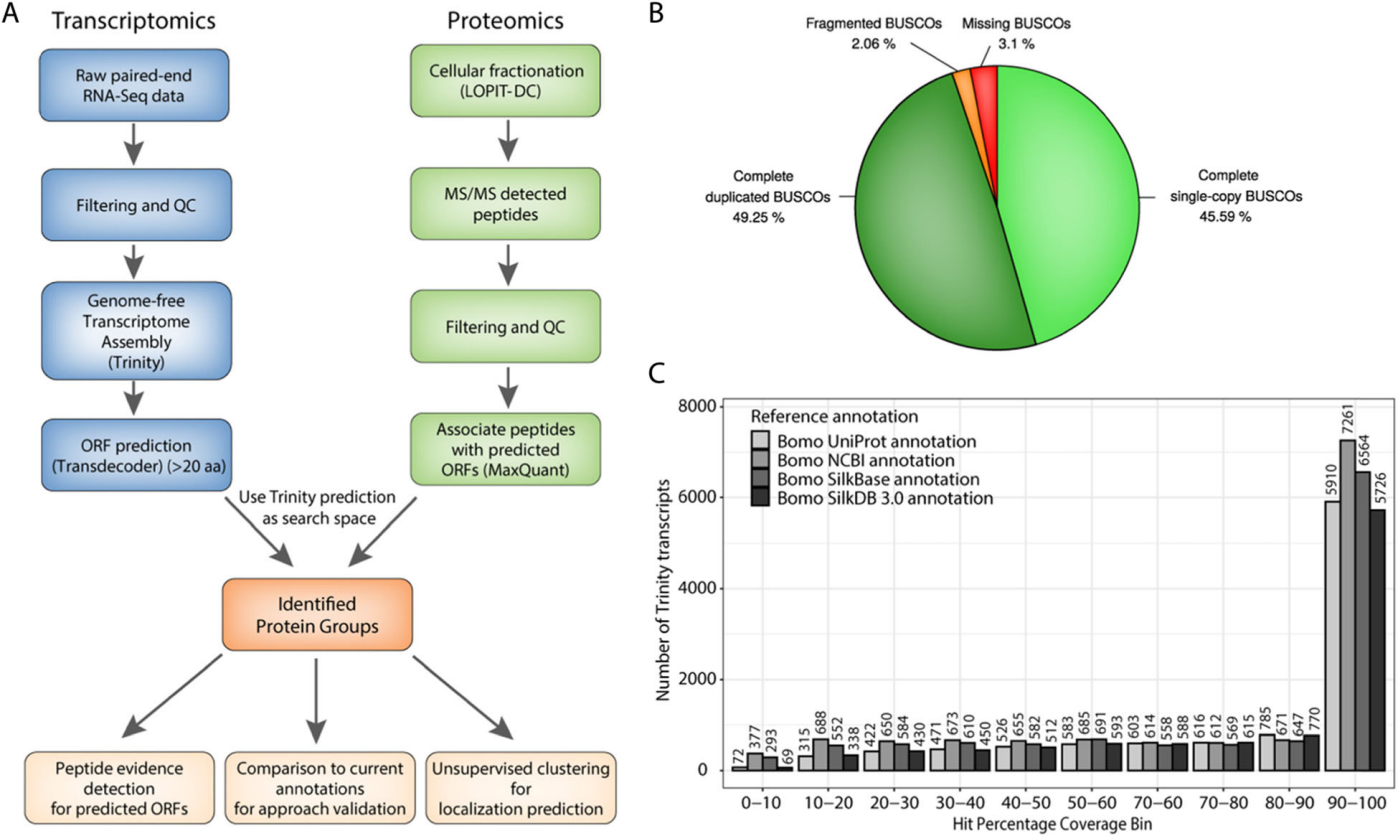
Genome-free transcriptome assembly approach and assessment of annotation quality. **a**. Overview of the proteotranscriptomics annotation approach. **b**. Pie-chart of BUSCO analysis based on the BUSCO arthropoda gene set. **c**. Barplot summarizing the results of a full-length transcript comparison between the genome-free Trinity assembly to currently available annotations from UniProt, NCBI, SilkBase and SilkDB 3.0

**Fig. 2:**
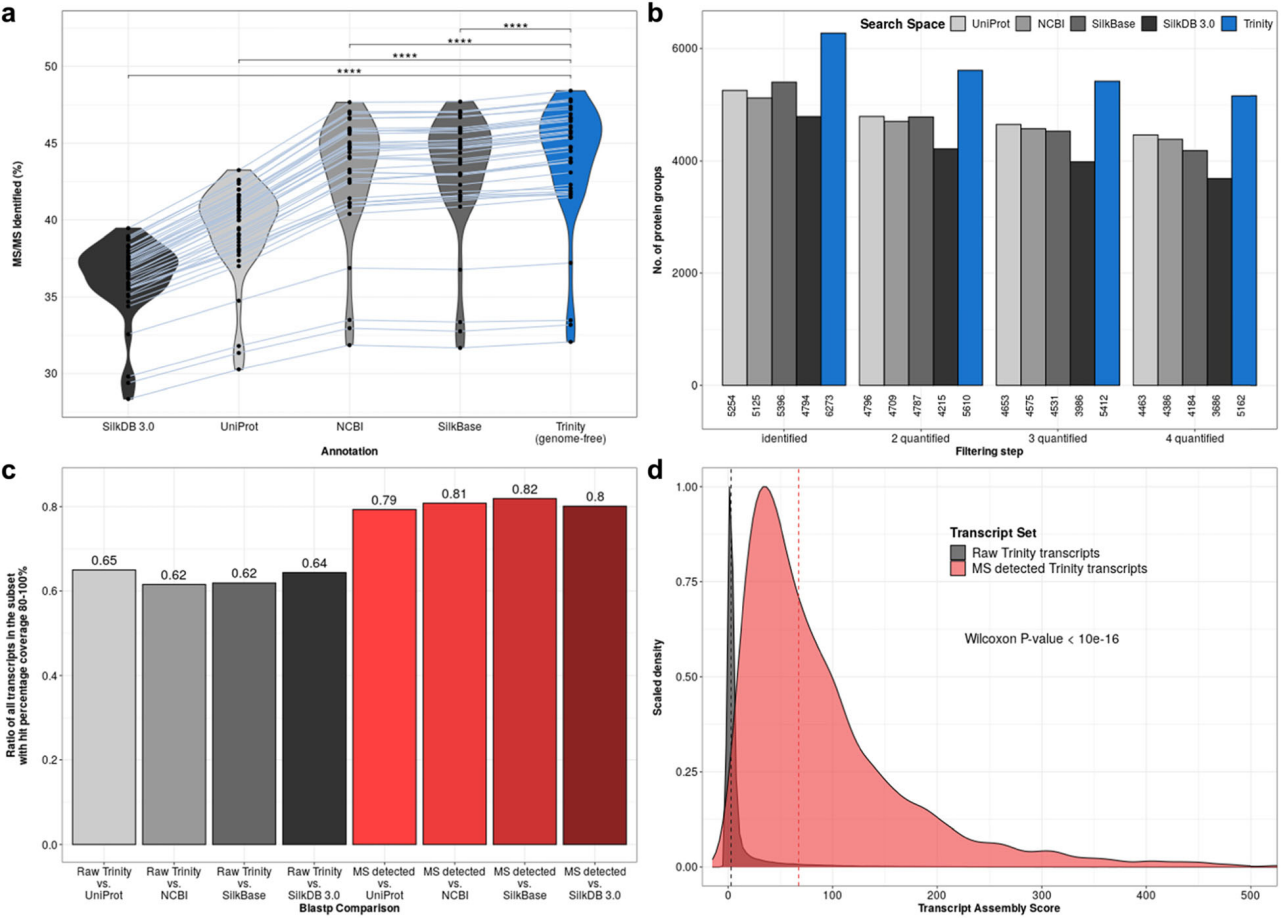
High resolution mass spectrometry provides evidence for superior genome-free annotation. **a**. Violin plots show distribution of identified MS/MS spectra (in percent) for each database used. With identical raw proteomic data the genome-free Trinity annotation shows significantly higher identified tandem MS spectra percentages than the four currently available annotations from UniProt, NCBI, SilkBase and SilkDB 3.0. Grey lines connect percentages stemming from the same MS run. **** indicates two-sided paired Wilcoxon signed rank test *p*-values below 0.0001. **b**. Barplot showing number of protein groups identified after different filtering steps with UniProt, NCBI, SilkBase, SilkDB 3.0 and genome-free Trinity annotation. The Trinity annotation shows higher numbers of identified protein groups for identification and quantification (including replicates). **c**. Barplot of the ratio of transcripts with a hit percentage coverage of more than 80% when compared to current *Bombyx mori* annotations. Grey bars include all Trinity annotated transcripts and red bars represent transcripts that have peptide evidences detected by MS. **d**. Scaled density plot showing distribution of transcript assembly scores of all Trinity annotated transcripts (gray) and transcripts with peptide evidences detected by MS (red). Dashed vertical lines indicate the median assembly score of each subset
