# Supplementary material for: Proteotranscriptomics assisted gene annotation and spatial proteomics of Bombyx mori BmN4 cell line
Source: BMC Genomics. 2020 Oct 6;21:690. doi: 10.1186/s12864-020-07088-7 (PMC7541253; doi:10.1186/s12864-020-07088-7)
Supplement: Supplementary file 1 — Additional file 1 Supplemental Fig. S1. Distribution of length distributions across RNA expression level bins. Supplemental Fig. S2. Comparison of Transrate assembly scores to other publicly available assemblies. Supplemental Fig. S3. Overall MS detected transcripts show improved assembly features. Supplemental Fig. S4. RNA-Seq coverage assay for the detection of falsely split genes. Supplemental Fig. S5. Densityplot of deviations from SilkBase annotation for longer annotated genes. Supplemental Fig. S6. Intracluster distances of SOM clusters can be used to filter out clusters that have high variability within the cluster. Supplemental Fig. S7. Depiction of expression profiles of all genes separated into respective clusters. Supplemental Fig. S8: Comparison of fractionation profiles of the respective cellular compartments in original LOPIT-DC TMT data and our Proteotranscriptomics LC-MS-MS approach. Supplemental Fig. S9. Comparison of fractionation profiles of orthologs of established Drosophila melanogaster cellular compartment markers to the mean dynamics determined by SOM clustering and enrichment analysis. Supplemental Fig. S10. Comparison of LFQ expression levels of detected protein groups that are shorter (small proteins) or longer than 20 amino acids. Supplemental Table S1. Read representation statistics of the Trinity assembly. Supplemental Table S2. Expression bins and transcript lenghts. Supplemental Table S3. TransRate analysis results. Supplemental Table S4. BUSCO analysis results. Supplemental Table S5. Bombyx mori NCBI and SilkBase based BmN4 variome. Supplemental Table S6. Statistics of correspondence and differences between genome-free and SilkBase annotations. Supplemental Table S7. Table including enrichment values for all clusters and all cellular localization categories. Supplemental Table S8. Mycoplasma contamination assay. Supplemental Table S9. Comparison of genome-free and genome-guided assembly. Supplemental Table S10. Mapping statistics of [file 12864_2020_7088_MOESM1_ESM.pdf]

Additional File 1

Proteotranscriptomics assisted gene annotation and spatial proteomics of the lepidopteran model species *Bombyx mori*

Michal Levin*, Marion Scheibe and Falk Butter*

Institute of Molecular Biology (IMB), Ackermannweg 4, 55128 Mainz, Germany

To whom correspondence should be addressed: m.levin@imb.de or f.butter@imb.de

Supplemental Figures:

Supplemental Figure S1: Distribution of length distributions across RNA expression level bins
Supplemental Figure S2: Comparison of Transrate assembly scores to other publicly available assemblies
Supplemental Figure S3: Overall MS detected transcripts show improved assembly features
Supplemental Figure S4: RNA-Seq coverage assay for the detection of falsely split genes
Supplemental Figure S5: Densityplot of deviations from SilkBase annotation for longer annotated genes
Supplemental Figure S6: Intracluster distances of SOM clusters can be used to filter out clusters that have high variability within the cluster
Supplemental Figure S7: Depiction of expression profiles of all genes separated into respective clusters
Supplemental Figure S8: Comparison of fractionation profiles of the respective cellular compartments in original LOPIT-DC TMT data and our Proteotranscriptomics LC-MS-MS approach
Supplemental Figure S9: Comparison of fractionation profiles of orthologs of established *Drosophila melanogaster* cellular compartment markers to the mean dynamics determined by SOM clustering and enrichment analysis
Supplemental Figure S10: Comparison of LFQ expression levels of detected protein groups that are shorter (small proteins) or longer than 20 amino acids

Supplemental Tables:

Supplemental Table S1: Read representation statistics of the Trinity assembly
Supplemental Table S2: Expression bins and transcript lenghts
Supplemental Table S3: TransRate analysis results
Supplemental Table S4: BUSCO analysis results
Supplemental Table S5: *Bombyx mori* NCBI and SilkBase based BmN4 variome
Supplemental Table S6: Statistics of correspondence and differences between genome-free and SilkBase annotations
Supplemental Table S7: Table including enrichment values for all clusters and all cellular localization categories
Supplemental Table S8: Mycoplasma contamination assay
Supplemental Table S9: Comparison of genome-free and genome-guided assembly
Supplemental Table S10. Mapping statistics of newly identified protein CDS sequences to the sequences of the female-determining chromosome W

Supplemental Figures

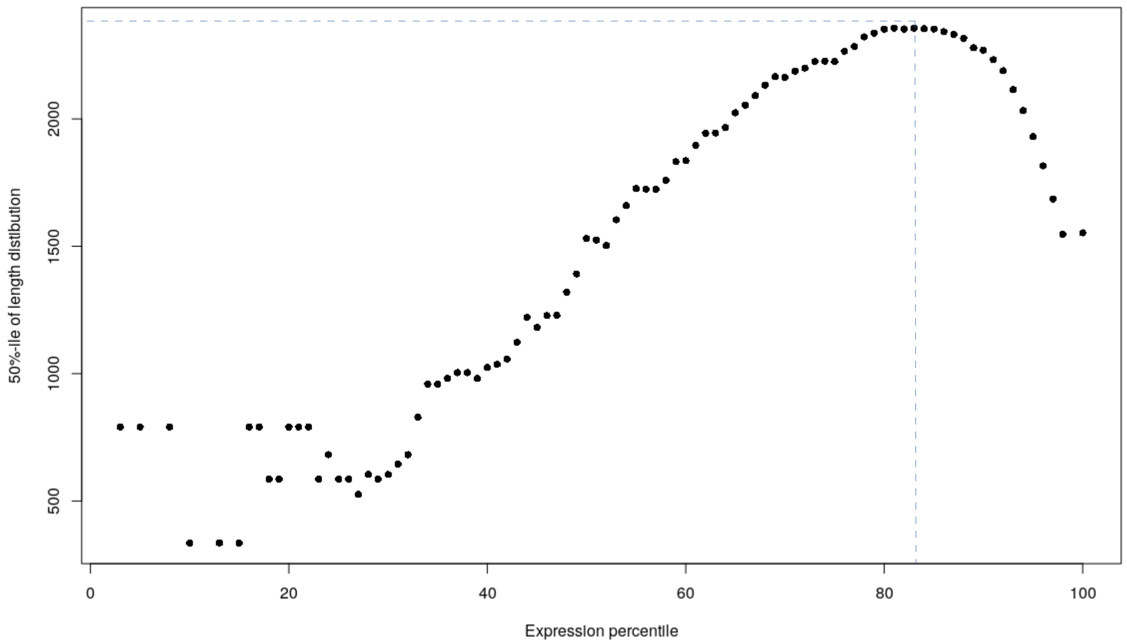

Supplemental Figure S1: Distribution of length distributions across RNA expression level bins. Trinity transcripts lengths (nt) peak at around the 80th percentile of expression levels of all individual transcripts (indicated by blue dotted line).

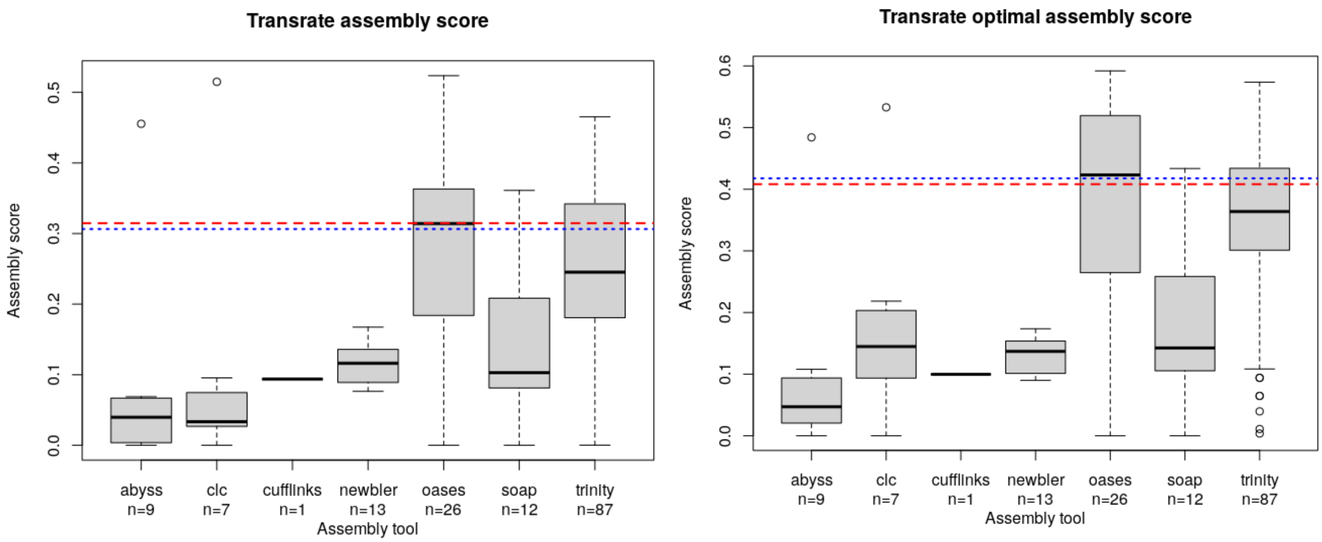

Supplemental Figure S2: Comparison of Transrate assembly scores to other publicly available assemblies. Transrate assembly scores of 255 assemblies analyzed by (Smith-Unna et al., 2016). Blue dotted horizontal lines mark the 70th percentile of the assembly or optimal assembly scores of all assemblies analyzed. Red dotted horizontal lines indicate the assembly or optimal assembly score of our trinity assembly.

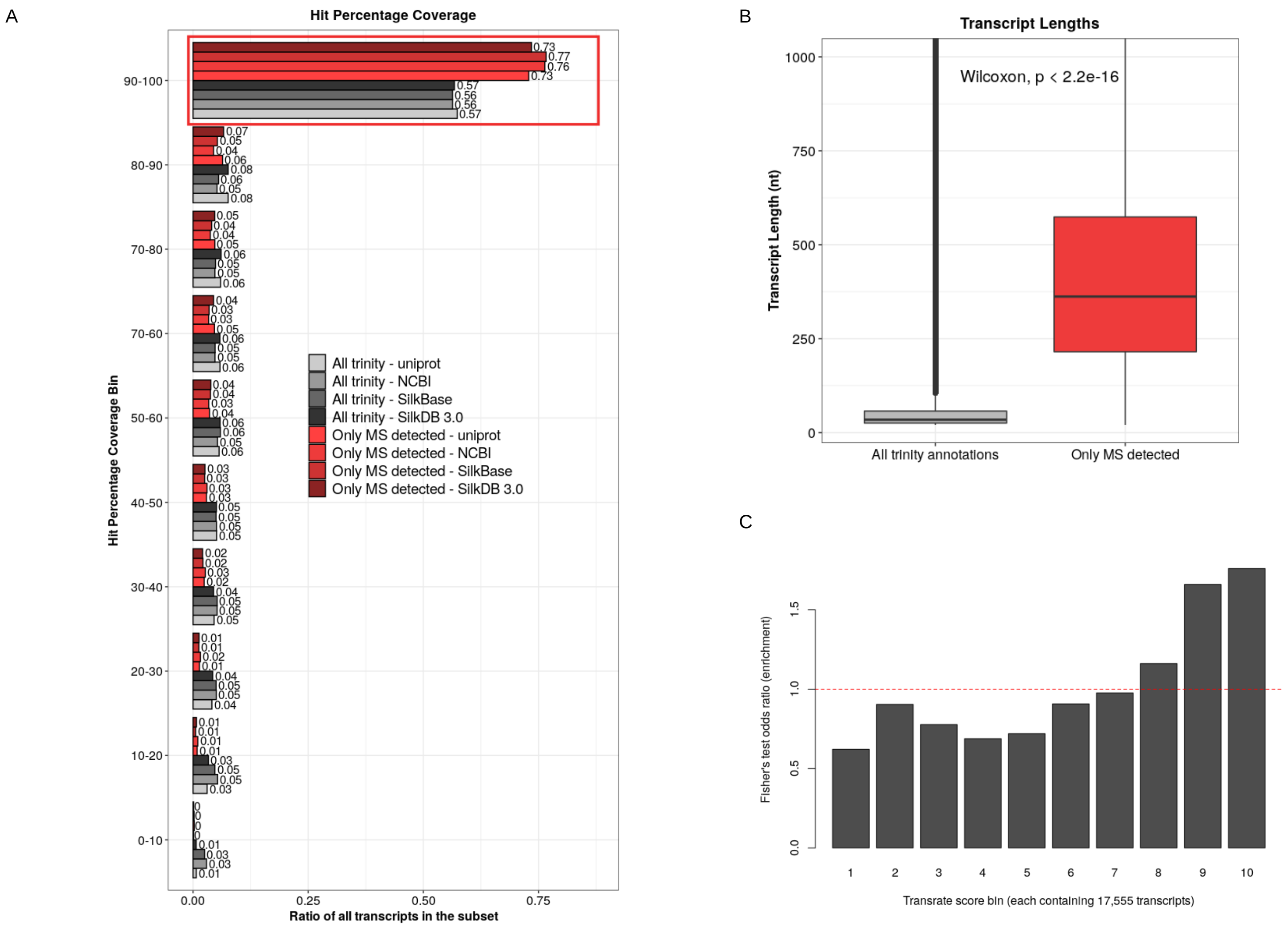

Supplemental Figure S3: Overall MS detected transcripts show improved assembly features.
(A) Barplot of hit percentage coverage bin of all transdecoder predictions and predictions that were detected by MS compared to current *Bombyx mori* annotations.(B) Boxplot of transcript lengths of all transdecoder predictions (grey) and predictions that were detected by mass spectrometry (MS) (red). (C) Barplot of mass spectrometric identification enrichment metrics across TransRate score bins. The plot shows that contigs with high TransRate scores tend to also be detected by mass spectrometry.

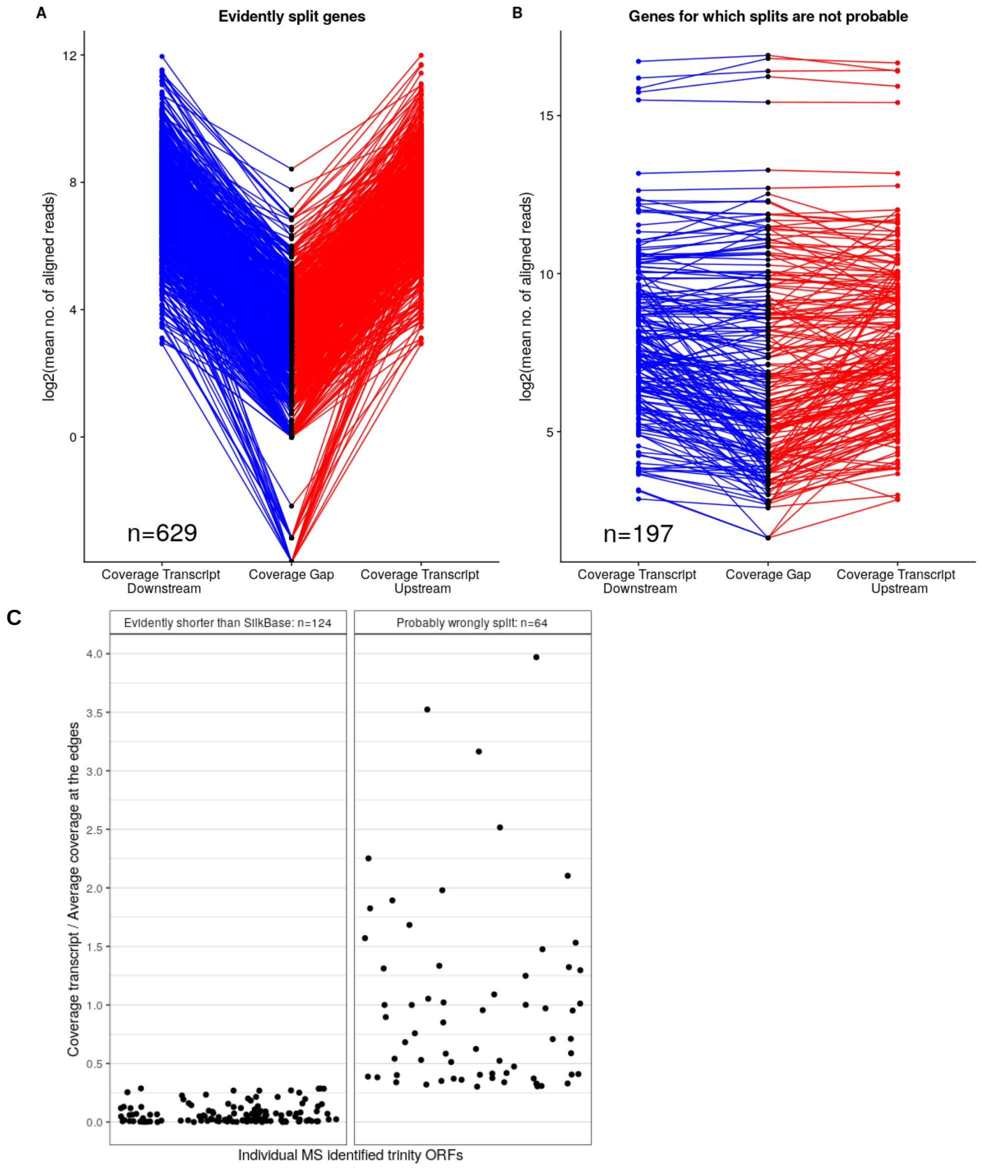

Supplemental Figure S4: RNA-Seq coverage assay for the detection of falsely split genes. For all SilkBase genes that were represented by split genes in the Trinity annotation the coverage for each transcript in the relevant paired transcripts (Transcript downstream and upstream – blue and red colored dots, respectively) and the gap between these (black colored dots) was calculated from mapped RNA-Seq paired reads. Blue and red lines indicate the change in coverage between downstream or upstream transcript and gap region, respectively. In general, one can distinguish two groups. (A) Transcripts that show a clear drop in coverage in the gap between the transcripts of interest and hence are most probably real splits. (B) Transcripts that show no significant drop in coverage in the gap region and hence could have in principle been falsely split in our approach. (C) 188 genes were found to be shorter than 85% compared to their SilkBase counterparts. 124 show a clear drop in the read coverage at the edges of the transcript (<30% of the gene body coverage), 64 don't show a decrease and are possibly falsely split).

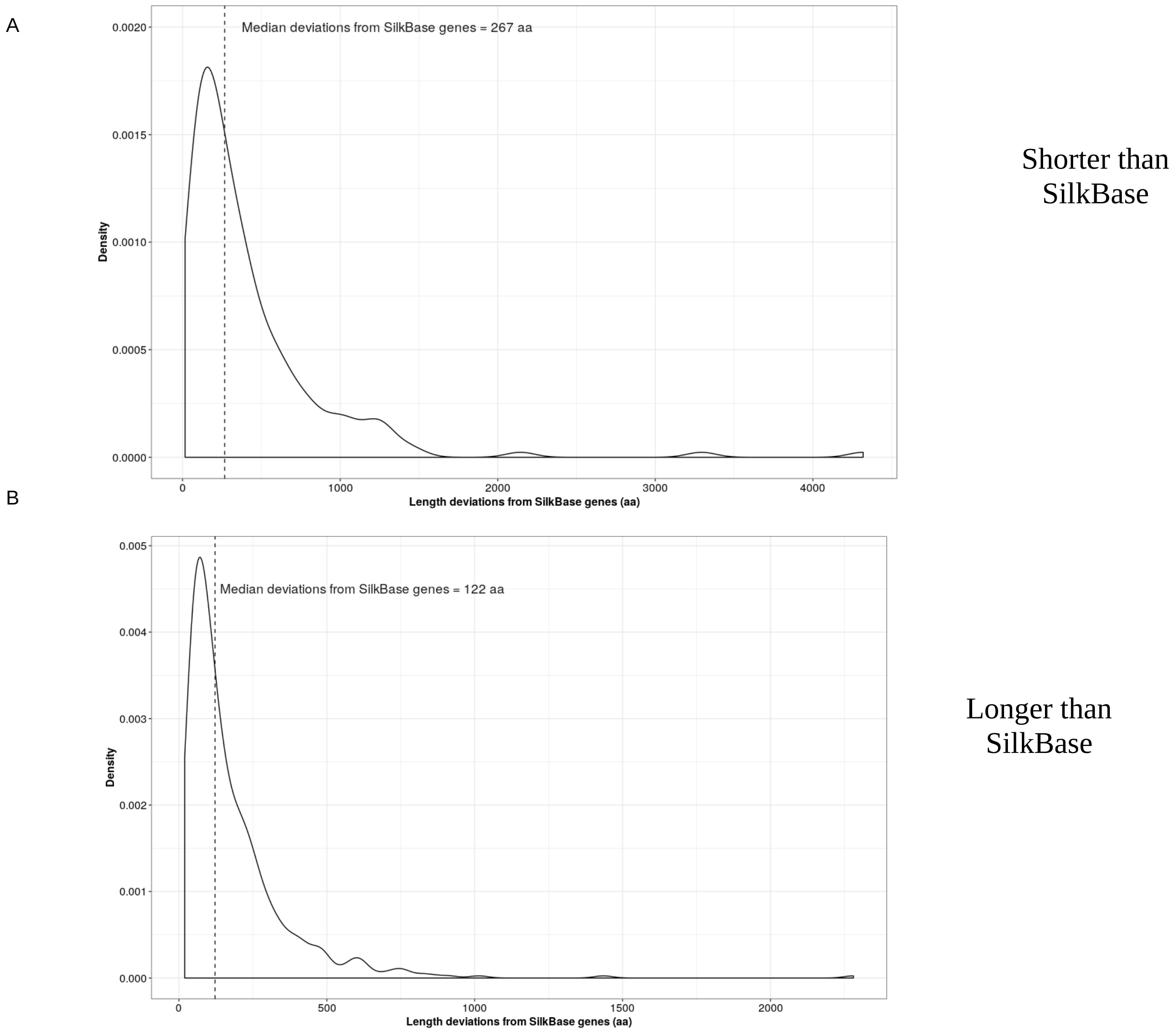

Supplemental Figure S5: Density plots describing the deviations of the protein lengths (amino acid residues) of MS detected Trinity assembled proteins from the lengths of associated SilkBase annotated proteins for proteins that have been identified to have (A) shorter assembled proteins (188 proteins in total) or (B) longer assembled proteins (513 proteins in total)

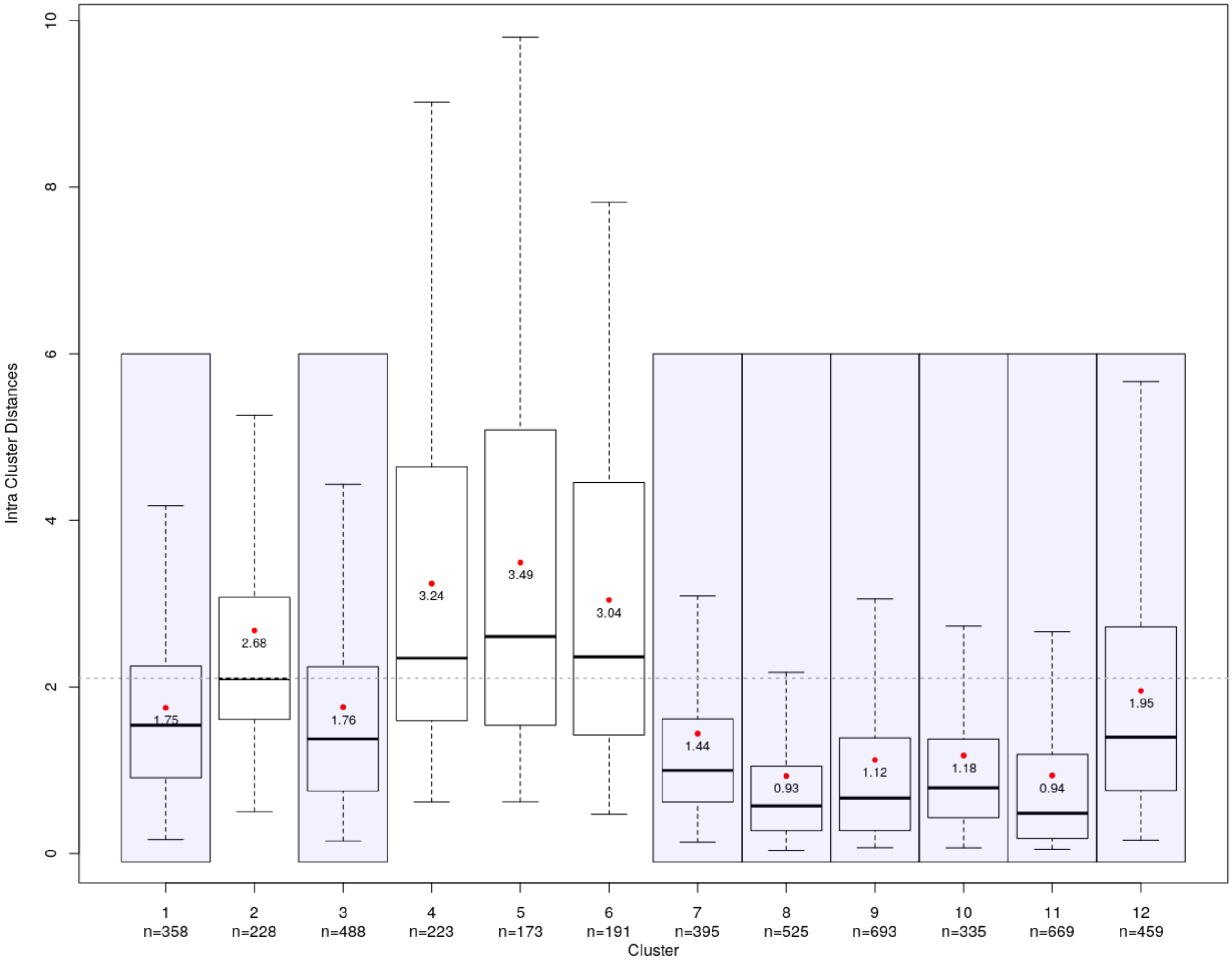

Supplemental Figure S6: Intracluster distances of SOM clusters can be used to filter out clusters that have high variability within the cluster (see Figure 4A). Boxplot summarizing the intra-cluster distances between fractionation profiles. For final analyses only clusters with mean intra-cluster distances (red dots and value shown) below the 75%-tile of all intra-cluster distances were kept (blue boxes). All others were combined into one cluster of uncategorized profiles (colored in gray in Fig. 4A).

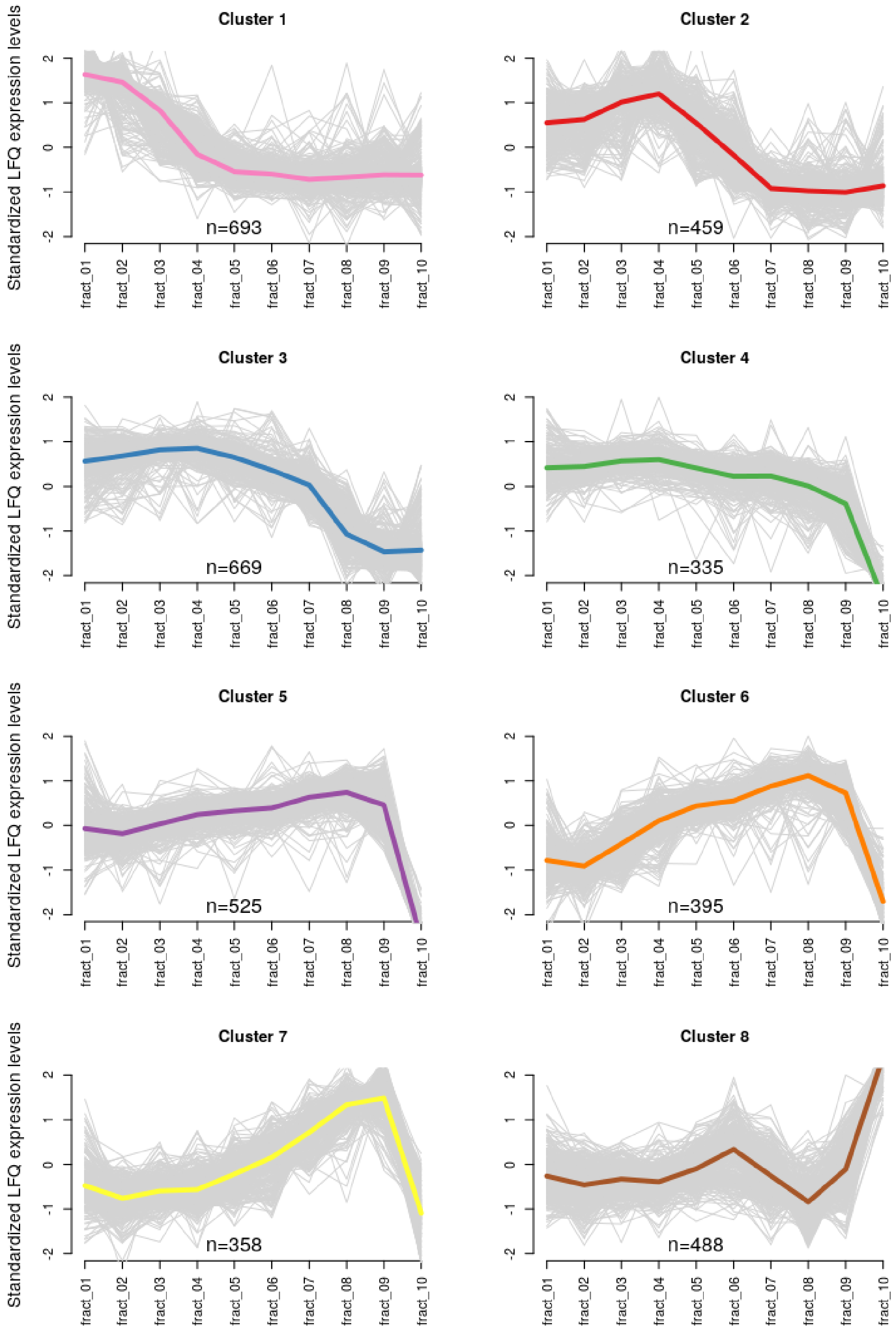

Supplemental Figure S7: Depiction of expression profiles of all genes separated into respective clusters (see Figure 4B). Individual standardized gene expression and average profiles across the fractionation series are depicted in thin gray and thick colored lines, respectively. Color code is the same as in Figure 4.

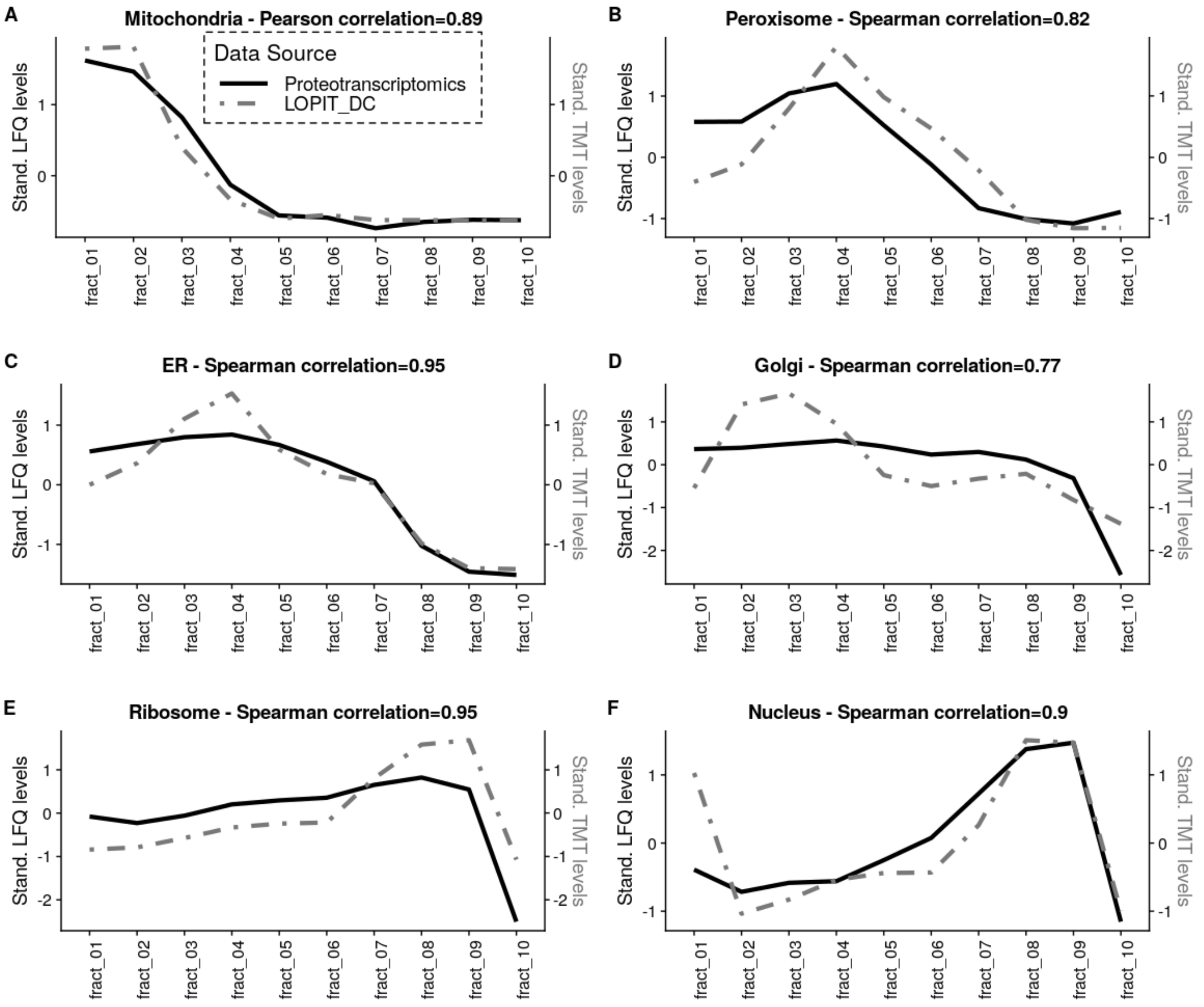

Supplemental Figure S8: Comparison of fractionation profiles of the respective cellular compartments show high correspondence between original LOPIT-DC TMT data and our Proteotranscriptomics LC-MS-MS approach. The black solid and the gray dashed line show the standardized mean LFQ levels from *Bombyx mori* BmN4 cells and the standardized mean TMT levels from human osteosarcoma U-2 OS cells of the respective clusters across fractions, respectively. Spearman correlation between the two experiments is indicated for each compartment in the title of the respective figure.

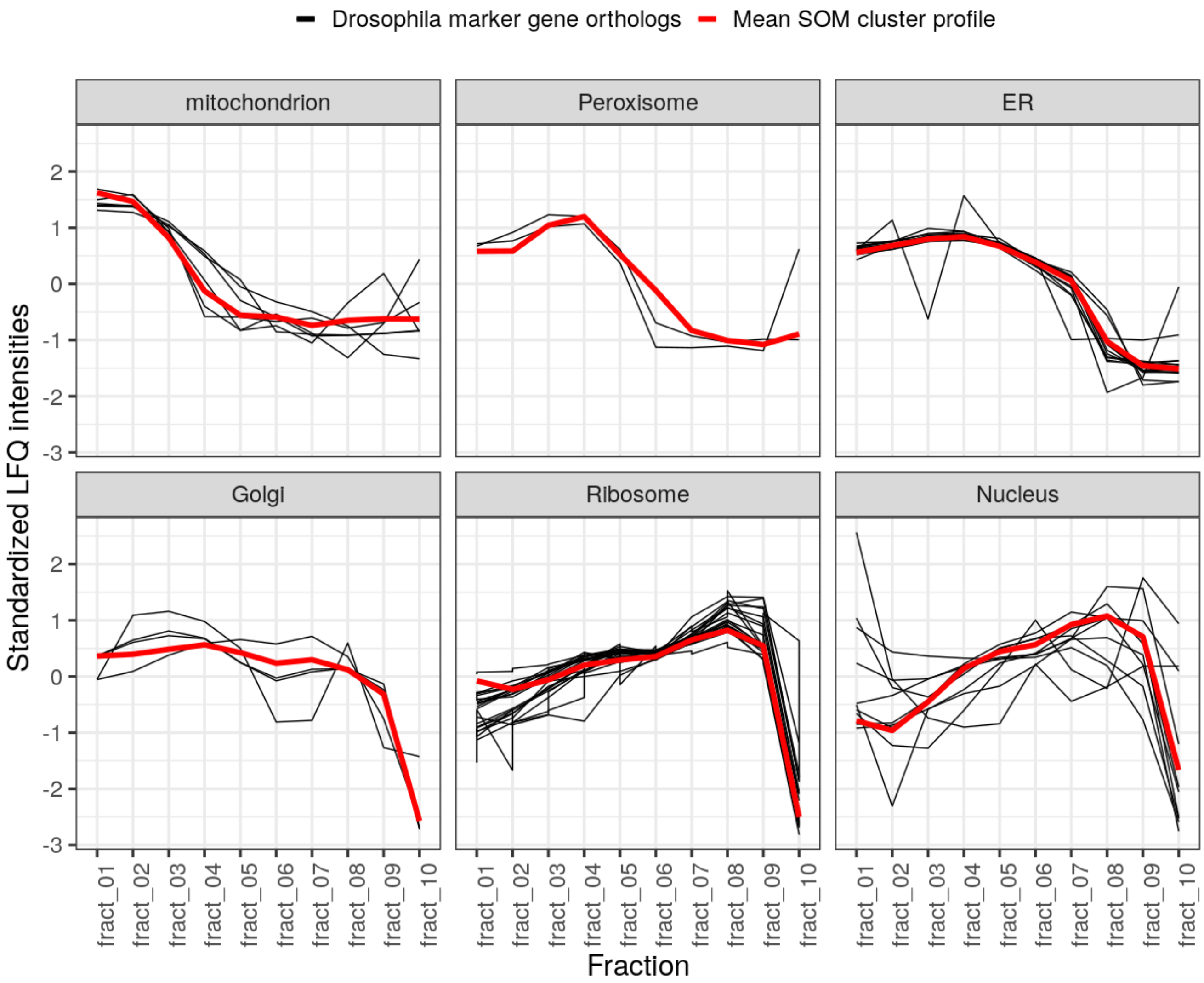

Supplemental Figure S9: Comparison of fractionation profiles of orthologs of established *Drosophila melanogaster* cellular compartment markers to the mean dynamics determined by SOM clustering and enrichment analysis show high correspondence. The black solid lines show the standardized LFQ levels of orthologous genes from *Bombyx mori* BmN4 cells. The thick red line shows standardized mean LFQ levels of the respective clusters as established in Figure 4B. Drosophila marker genes for cellular compartments were retrieved from pRoloc bioconductor package (PMID: 24413670), orthology was established using blastp. ER: Q8T9B6, Q8STG9, Q9VUZ0, Q9U5L1, Q24319, Q9VIU7, Q8T045, Q09332, O18405, Q9VSU7, Q9VSN9, Q7KLX3, Q7K110. Golgi: Q9VR90, Q6WV19, Q6WV17, Q9VYV5. Mitochondrion: Q9VEJ0, P35381, Q9VSR5, Q9VIE8, Q95RF6, Q94516. Nucleus: O44437, Q24492, Q8IPX7, P27864, P15348, Q9W1V3, Q9V3E7, P41073, A1Z8U0, A1ZBW0. Peroxisome: P17336, Q9VUL8. Ribosome: P41094, P50887, Q9VVU2, P41093, Q9VTP4, P48159, Q9W229, P38979, P38979, Q9V9M7, P55841, P55830, P04359, P80455, O16797, P29327, P09180, P09180, Q9W1B9, Q9V3G1, Q9V3G1, P17704, Q0E9B6, Q0E9B6, Q9VNB9

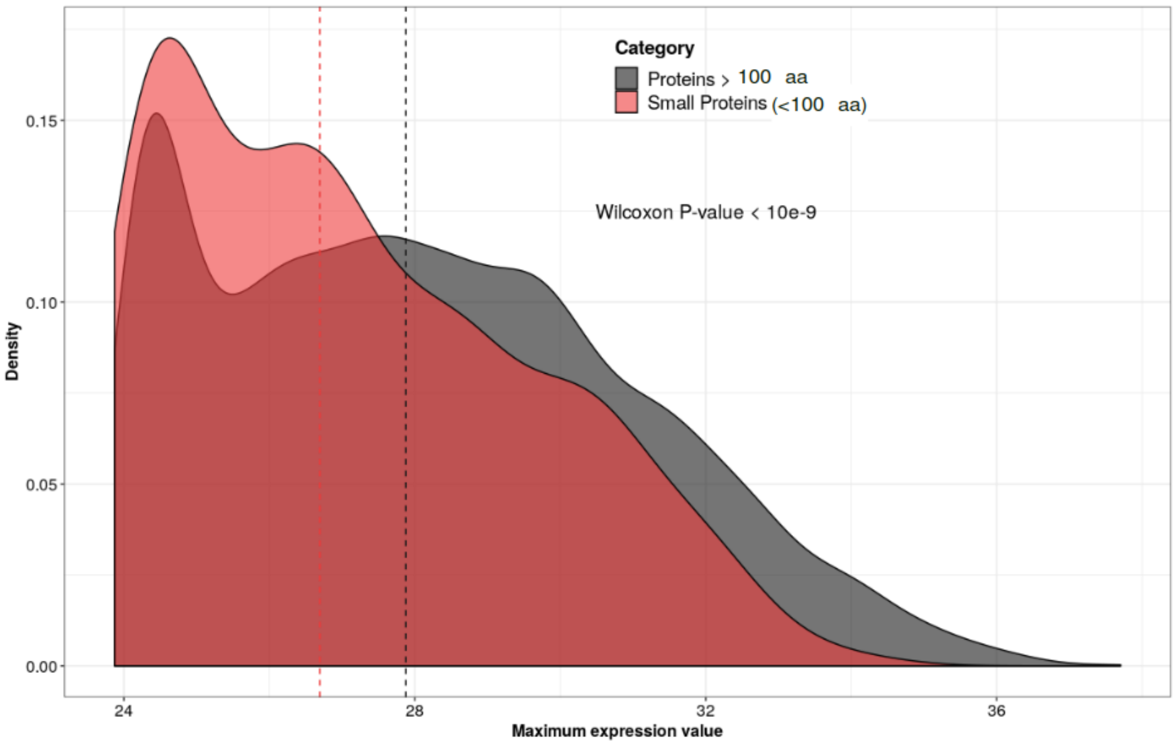

Supplemental Figure S10: Comparison of LFQ expression levels of detected protein groups that are shorter (small proteins n=308) or longer than 100 amino acids (n=5965). Density plot describes the distribution of maximum protein expression levels detected in our assay.

Supplemental Table S1: Read representation statistics of the Trinity assembly

General bowtie2 alignment statistics

164204260 reads; of these:
164,204,260 (100.00%) were paired; of these:
6,910,436 (4.21%) aligned concordantly 0 times
32,754,566 (19.95%) aligned concordantly exactly 1 time
124,539,258 (75.84%) aligned concordantly >1 times

6,910,436 pairs aligned concordantly 0 times; of these:
222,380 (3.22%) aligned discordantly 1 time

6,688,056 pairs aligned 0 times concordantly or discordantly; of these:
13,376,112 mates make up the pairs; of these:
5,520,530 (41.27%) aligned 0 times
1,518,998 (11.36%) aligned exactly 1 time
6,336,584 (47.37%) aligned >1 times

98.32% overall alignment rate

Stats for aligned rna-seq fragments (note, not counting those frags where neither left/right read aligned)

162139814 aligned fragments; of these:
162139814 were paired; of these:
4845990 aligned concordantly 0 times
157293824 aligned concordantly exactly 1 time
0 aligned concordantly >1 times

4845990 pairs aligned concordantly 0 times; of these:
3454352 aligned as improper pairs
1391638 pairs had only one fragment end align to one or more contigs; of these:
1092884 fragments had only the left /1 read aligned; of these:
1092884 left reads mapped uniquely
0 left reads mapped >1 times
298754 fragments had only the right /2 read aligned; of these:
298754 right reads mapped uniquely
0 right reads mapped >1 times

Overall, 97.01% of aligned fragments aligned as proper pairs

Supplemental Table S2: Expression bins and transcripts lenghts

Expression percentile	ExN50 (median length (bases) of transcripts in expression perc bin)	# transcripts
3	791	1
5	791	2
8	791	3
10	335	4
13	335	5
15	335	6
16	791	7
17	791	8
18	586	10
19	586	11
20	791	13
21	791	15
22	791	18
23	586	20
24	682	23
25	586	26
26	586	30
27	526	33
28	604	37
29	586	41
30	604	45
31	645	49
32	682	54
33	829	59
34	959	64
35	959	70
36	981	76
37	1004	83
38	1004	90
39	981	98
40	1024	106
41	1037	115
42	1057	125
43	1123	137
44	1221	149
45	1181	163
46	1228	178
47	1229	195
48	1320	215
49	1392	238
50	1530	264
51	1525	295
52	1503	330
53	1604	370
54	1660	416

Expression percentile	ExN50 (median length (bases) of transcripts in expression perc group)	# transcripts
55	1727	472
56	1724	535
57	1724	609
58	1759	692
59	1832	785
60	1836	890
61	1896	1008
62	1944	1141
63	1945	1289
64	1966	1456
65	2024	1641
66	2054	1844
67	2092	2068
68	2132	2315
69	2166	2588
70	2163	2889
71	2188	3221
72	2200	3590
73	2225	3993
74	2227	4439
75	2225	4930
76	2266	5471
77	2284	6071
78	2322	6737
79	2337	7476
80	2352	8297
81	2356	9212
82	2353	10244
**83**	**2356**	**11414**
84	2354	12751
85	2353	14291
86	2342	16072
87	2332	18181
88	2317	20718
89	2279	23817
**90**	**2270**	**27688**
91	2232	32659
92	2189	39193
93	2115	47777
94	2033	58804
95	1931	72524
96	1816	89225
97	1686	109447
98	1547	134574
100	1553	186401

Supplemental Table S3. TransRate analysis results

**assembly**	Trinity.fasta
**n_seqs**	186,401
**smallest**	201
**largest**	20,434
**n_bases**	158,589,380
**mean_len**	850.80
**n_under_200**	0
**n_over_1k**	44,509
**n_over_10k**	103
**n_with_orf**	34,345
**mean_orf_percent**	45.31
**n90**	317
**n70**	761
**n50**	1,553
**n30**	2,608
**n10**	4,771
**gc**	0.39
**bases_n**	0
**proportion_n**	0
**fragments**	164,204,260
**fragments_mapped**	140,021,124
**p_fragments_mapped**	0.85
**good_mappings**	119,452,817
**p_good_mapping**	0.73
**bad_mappings**	20,568,307
**potential_bridges**	78,186
**bases_uncovered**	21,535,350
**p_bases_uncovered**	0.14
**contigs_uncovbase**	86,782
**p_contigs_uncovbase**	0.47
**contigs_uncovered**	16,022
**p_contigs_uncovered**	0.09
**contigs_lowcovered**	117,167
**p_contigs_lowcovered**	0.63
**contigs_segmented**	15,615
**p_contigs_segmented**	0.08
**CRBB_hits**	33,518
**n_contigs_with_CRBB**	33,518
**p_contigs_with_CRBB**	0.18
**rbh_per_reference**	1.49
**n_refs_with_CRBB**	12,564
**p_refs_with_CRBB**	0.56
**cov25**	11,476
**p_cov25**	0.51
**cov50**	10,224
**p_cov50**	0.45
**cov75**	8,756
**p_cov75**	0.39
**cov85**	8,040
**p_cov85**	0.36
**cov95**	7,190
**p_cov95**	0.32
**reference_coverage**	0.44
**score**	**0.31**
**optimal_score**	0.41
**cutoff**	0.04
**weighted**	0.54

Supplemental Table S4. BUSCO analysis results
BUSCO version is: 2.0 beta 2
The lineage dataset is: arthropoda_odb9
BUSCO was run in mode: proteins

C:94.8%[S:45.6%,D:49.2%],F:2.1%,M:3.1%,n:1066

1011	Complete BUSCOs (C)
486	Complete and single-copy BUSCOs (S)
525	Complete and duplicated BUSCOs (D)
22	Fragmented BUSCOs (F)
33	Missing BUSCOs (M)
1066	Total BUSCO groups searched

Supplemental Table S5. *Bombyx mori* NCBI-BmN4 and SilkBase-BmN4 variome

**Genome**	CDS *Bombyx mori* NCBI	CDS *Bombyx mori* SilkBase
**SnpEff version**	SnpEff 4.3t	SnpEff 4.3t
**Number of lines (input file)**	125,939	186,952
**Number of multi-allelic VCF entries (i.e. more than two alleles)**	361	582
**Number of effects**	126,300	187,534
**Genome total length**	36,783,937	26,184,828
**Genome effective length**	16,393,027	19,826,985
**Variant rate**	1 variant every 129 bases	1 variant every 105 bases

Number variants by type

	NCBI		SilkBase	
**Type**	**Total**	**Percent**	**Total**	**Percent**
**SNP**	124,041	98.21%	184,303	98.28%
**MNP**	0	0.00%	0	0.00%
**INS**	1,287	1.02%	1,547	0.82%
**DEL**	972	0.77%	1,684	0.90%
**MIXED**	0	0.00%	0	0.00%
**INV**	0	0.00%	0	0.00%
**DUP**	0	0.00%	0	0.00%
**BND**	0	0.00%	0	0.00%
**INTERVAL**	0	0.00%	0	0.00%
**Sum**	126,300		187,534	

Number of effects by impact

	NCBI		SilkBase	
**Type (alphabetical order)**	**Count**	**Percent**	**Count**	**Percent**
**HIGH**	2,053	1.62%	2,986	1.59%
**LOW**	93,155	73.75%	136,664	72.87%
**MODERATE**	31,092	24.61%	47,884	25.53%
Sum	126,300		187,534	

Number of effects of SNP by functional class

	NCBI		SilkBase	
**Type (alphabetical order)**	**Count**	**Percent**	**Count**	**Percent**
**MISSENSE**	30,541	24.62%	46,793	25.39%
**NONSENSE**	345	0.28%	846	0.46%
**SILENT**	93,155	75.1%	136,664	74.15%
Sum	124,041		184,303	

Number of effects by type and region

	NCBI		SilkBase	
**Type (alphabetical order)**	**Count**	**Percent**	**Count**	**Percent**
**conservative_inframe_deletion**	124	0.09%	219	0.12%
**conservative_inframe_insertion**	92	0.07%	181	0.1%
**disruptive_inframe_deletion**	225	0.17%	401	0.21%
**disruptive_inframe_insertion**	176	0.13%	323	0.17%
**frameshift_variant**	1,642	1.29%	2,107	1.12%
**missense_variant**	30,484	24.12%	46,774	24.93%
**splice_region_variant**	10	0.008%	17	0.01%
**stop_gained**	395	0.31%	918	0.49%
**stop_lost**	62	0.05%	19	0.01%
**synonymous_variant**	93,146	73.71%	136,664	72.84%

Supplemental Table S6. Statistics of correspondence and differences between genome-free (BOMOPTA) and SilkBase annotations

Total # of instances with potential split
(blastp results BOMOPTA vs. SilkBase retrieve more than one result with minimally overlapping BOMOTPA transcripts)

941	BOMOPTA Gene pairs
631	SilkBase Genes

Overlapping transcripts
(transcripts overlap by at least 100 bp)

110	BOMOPTA Gene pairs
101	SilkBase Genes

Consecutive Genes
(no overlap between transcripts)

823	BOMOPTA Gene pairs
561	SilkBase Genes

Different strand
(gene pairs map to different strands)

6	BOMOPTA Gene pairs
6	SilkBase Genes

Different chromosome
(gene pairs map to different chromosomes)

2	BOMOPTA Gene pairs
2	SilkBase Genes

Evidently split genes
(clear coverage decrease of RNA-Seq reads in gap regions)

**628**	BOMOPTA Gene pairs
451	SilkBase Genes

Erroneous split
(no coverage decrease of RNA-Seq reads in gap regions)

**195**	BOMOTPA Gene pairs
161	SilkBase Genes

Supplemental Table S7. Table including enrichment values for all clusters and all cellular localization categories

Cluster_ID	Annotation_Category	No. genes in cluster and category	No. genes not in cluster and in category	No. genes in cluster and not in category	No. genes not in cluster and not in category	p.value	fold_change	p.value_adj
cluster_1	TmHMM	124	618	575	3423	5.8E-02	1.19	2.3E-01
cluster_1	signalP	13	268	686	3773	1.0E+00	0.27	1.0E+00
cluster_1	Lysosome_cc	3	44	696	3997	9.8E-01	0.39	1.0E+00
cluster_1	Peroxisome_cc	3	27	696	4014	8.4E-01	0.64	1.0E+00
cluster_1	Golgi_cc	2	108	697	3933	1.0E+00	0.10	1.0E+00
cluster_1	Nucleus_cc	20	658	679	3383	1.0E+00	0.15	1.0E+00
cluster_1	Chromatin_cc	6	48	693	3993	8.3E-01	0.72	1.0E+00
cluster_1	ER_cc	1	96	698	3945	1.0E+00	0.06	1.0E+00
cluster_1	Mitochondrion_cc	301	84	398	3957	5.1E-197	35.56	4.1E-195
cluster_1	Ribosomes	2	81	697	3960	1.0E+00	0.14	1.0E+00
cluster_2	TmHMM	212	530	291	3707	7.2E-53	5.09	1.9E-51
cluster_2	signalP	103	178	400	4059	1.3E-33	5.87	2.0E-32
cluster_2	Lysosome_cc	15	32	488	4205	5.9E-05	4.04	3.4E-04
cluster_2	Peroxisome_cc	22	8	481	4229	6.3E-16	24.14	6.3E-15
cluster_2	Golgi_cc	15	95	488	4142	1.9E-01	1.34	6.2E-01
cluster_2	Nucleus_cc	17	661	486	3576	1.0E+00	0.19	1.0E+00
cluster_2	Chromatin_cc	4	50	499	4187	8.4E-01	0.67	1.0E+00
cluster_2	ER_cc	26	71	477	4166	5.0E-06	3.20	3.3E-05
cluster_2	Mitochondrion_cc	12	373	491	3864	1.0E+00	0.25	1.0E+00
cluster_2	Ribosomes	1	82	502	4155	1.0E+00	0.10	1.0E+00
cluster_3	TmHMM	312	430	337	3661	8.0E-103	7.88	3.2E-101
cluster_3	signalP	135	146	514	3945	2.2E-47	7.09	4.4E-46
cluster_3	Lysosome_cc	13	34	636	4057	8.5E-03	2.44	4.0E-02
cluster_3	Peroxisome_cc	2	28	647	4063	9.3E-01	0.45	1.0E+00
cluster_3	Golgi_cc	23	87	626	4004	2.3E-02	1.69	9.6E-02
cluster_3	Nucleus_cc	22	656	627	3435	1.0E+00	0.18	1.0E+00
cluster_3	Chromatin_cc	3	51	646	4040	9.8E-01	0.37	1.0E+00
cluster_3	ER_cc	33	64	616	4027	2.2E-07	3.37	1.6E-06
cluster_3	Mitochondrion_cc	3	382	646	3709	1.0E+00	0.05	1.0E+00
cluster_3	Ribosomes	1	82	648	4009	1.0E+00	0.08	1.0E+00
cluster_4	TmHMM	57	685	310	3688	5.5E-01	0.99	1.0E+00
cluster_4	signalP	15	266	352	4107	9.6E-01	0.66	1.0E+00
cluster_4	Lysosome_cc	4	43	363	4330	5.0E-01	1.11	1.0E+00
cluster_4	Peroxisome_cc	1	29	366	4344	9.1E-01	0.41	1.0E+00
cluster_4	Golgi_cc	21	89	346	4284	8.0E-05	2.92	4.3E-04
cluster_4	Nucleus_cc	46	632	321	3741	8.6E-01	0.85	1.0E+00
cluster_4	Chromatin_cc	3	51	364	4322	8.0E-01	0.70	1.0E+00
cluster_4	ER_cc	6	91	361	4282	7.7E-01	0.78	1.0E+00
cluster_4	Mitochondrion_cc	12	373	355	4000	1.0E+00	0.36	1.0E+00
cluster_4	Ribosomes	1	82	366	4291	1.0E+00	0.14	1.0E+00
cluster_5	TmHMM	8	734	504	3494	1.0E+00	0.08	1.0E+00
cluster_5	signalP	2	279	510	3949	1.0E+00	0.06	1.0E+00
cluster_5	Lysosome_cc	5	42	507	4186	5.8E-01	0.98	1.0E+00
cluster_5	Peroxisome_cc	1	29	511	4199	9.7E-01	0.28	1.0E+00
cluster_5	Golgi_cc	17	93	495	4135	8.0E-02	1.53	3.1E-01
cluster_5	Nucleus_cc	107	571	405	3657	1.1E-05	1.69	6.5E-05
cluster_5	Chromatin_cc	9	45	503	4183	1.2E-01	1.66	4.5E-01
cluster_5	ER_cc	10	87	502	4141	6.1E-01	0.95	1.0E+00
cluster_5	Mitochondrion_cc	10	375	502	3853	1.0E+00	0.20	1.0E+00
cluster_5	Ribosomes	47	36	465	4192	5.5E-25	11.76	7.3E-24
cluster_6	TmHMM	2	740	380	3618	1.0E+00	0.03	1.0E+00
cluster_6	signalP	3	278	379	4080	1.0E+00	0.12	1.0E+00
cluster_6	Lysosome_cc	2	45	380	4313	9.0E-01	0.50	1.0E+00
cluster_6	Peroxisome_cc	1	29	381	4329	9.2E-01	0.39	1.0E+00
cluster_6	Golgi_cc	11	99	371	4259	2.7E-01	1.28	8.7E-01
cluster_6	Nucleus_cc	108	570	274	3788	8.4E-14	2.62	7.5E-13
cluster_6	Chromatin_cc	11	43	371	4315	3.2E-03	2.97	1.6E-02
cluster_6	ER_cc	6	91	376	4267	8.1E-01	0.75	1.0E+00
cluster_6	Mitochondrion_cc	3	382	379	3976	1.0E+00	0.08	1.0E+00
cluster_6	Ribosomes	30	53	352	4305	3.4E-13	6.92	2.7E-12
cluster_7	TmHMM	3	739	350	3648	1.0E+00	0.04	1.0E+00
cluster_7	signalP	6	275	347	4112	1.0E+00	0.26	1.0E+00
cluster_7	Lysosome_cc	2	45	351	4342	8.8E-01	0.55	1.0E+00
cluster_7	Peroxisome_cc	1	29	352	4358	9.0E-01	0.43	1.0E+00
cluster_7	Golgi_cc	4	106	349	4281	9.7E-01	0.46	1.0E+00
cluster_7	Nucleus_cc	113	565	240	3822	6.7E-19	3.18	7.6E-18
cluster_7	Chromatin_cc	9	45	344	4342	1.7E-02	2.52	7.5E-02
cluster_7	ER_cc	4	93	349	4294	9.4E-01	0.53	1.0E+00
cluster_7	Mitochondrion_cc	6	379	347	4008	1.0E+00	0.18	1.0E+00
cluster_7	Ribosomes	2	81	351	4306	9.9E-01	0.30	1.0E+00
cluster_8	TmHMM	7	735	485	3513	1.0E+00	0.07	1.0E+00
cluster_8	signalP	3	278	489	3970	1.0E+00	0.09	1.0E+00
cluster_8	Lysosome_cc	6	41	486	4207	3.6E-01	1.27	1.0E+00
cluster_8	Peroxisome_cc	4	26	488	4222	3.8E-01	1.33	1.0E+00
cluster_8	Golgi_cc	7	103	485	4145	9.5E-01	0.58	1.0E+00
cluster_8	Nucleus_cc	79	599	413	3649	1.4E-01	1.17	4.7E-01
cluster_8	Chromatin_cc	3	51	489	4197	9.3E-01	0.50	1.0E+00
cluster_8	ER_cc	6	91	486	4157	9.5E-01	0.56	1.0E+00
cluster_8	Mitochondrion_cc	21	364	471	3884	1.0E+00	0.48	1.0E+00
cluster_8	Ribosomes	2	81	490	4167	1.0E+00	0.21	1.0E+00

Supplemental Table S8. Mycoplasma contamination assay

Mycoplasma species	No. of Q20 mapped reads	No. of the M. mapped reads that re-map to SilkBase bombyx mori genome	Percentage of 160 M reads
A.laidlawii	7	0	0.0000043750
M.arginini	97	0	0.0000606250
M.fermentans	427	0	0.0002668750
M.hominis	0	0	0.0000000000
M.hyorinis	256	0	0.0001600000
M.orale	94	0	0.0000587500
All Mycoplasma species combined	223	0	0.0001393750

Supplemental Table S9. Comparison of genome-free and genome-guided assembly

Assemblies were performed using the same RNA-Seq and mass spectrometry data. For the genome-guided approach the SilkBase genome was used as scaffold for read mapping.

Parameter	Genome-free assembly	Genome-guided assembly
**Raw Trinity Assembly**		
**Read to assembled contigs alignment rate**	98.32%	56.95%
**Mean length of contigs**	850 bases	793 bases
**No. of good contigs (transrate)**	172,778	160,865
**% complete BUSCOs**	94.84%	93.60%
**# contigs with refs in silkbase (CRBB)**	12564 (56%)	12072 (54%)
**% contigs with sequence coverage > 85%**	36%	34%
**Mass-Spectrometric Identification**		
**Total MS/MS identified**	43.98%	44.11%
**# protein groups identified**	6,273	6,125
**% identified ORFs with > 80% hit percentage (genome-free vs. genome-guided)**	96%	
**> 80% hit percentage (genome-free vs. genome-guided)**	220 (4%)	232 (4%)

Supplemental Table S10. Mapping statistics of newly identified protein CDS sequences to the sequences of the female-determining chromosome W [

Chromosome	No. of newly identified genes mapping to respective chromosome	Length (bp)	Gene per bp
BMSK_chr1	2	21484951	9.31E-08
BMSK_chr2	4	8686177	2.30E-07
BMSK_chr3	6	15247601	1.31E-07
BMSK_chr4	3	19249693	1.04E-07
BMSK_chr5	9	19440377	1.03E-07
BMSK_chr6	5	17184971	1.16E-07
BMSK_chr7	4	14081139	1.42E-07
BMSK_chr8	7	16207405	1.23E-07
BMSK_chr9	6	17323741	1.15E-07
BMSK_chr10	5	17823796	1.12E-07
BMSK_chr11	16	20585495	9.72E-08
BMSK_chr12	10	17604390	1.14E-07
BMSK_chr13	5	17827900	1.12E-07
BMSK_chr14	2	13351588	1.50E-07
BMSK_chr15	12	18484841	1.08E-07
BMSK_chr16	7	14645837	1.37E-07
BMSK_chr17	6	18863447	1.06E-07
BMSK_chr18	3	16364595	1.22E-07
BMSK_chr19	5	14821947	1.35E-07
BMSK_chr20	4	12398063	1.61E-07
BMSK_chr21	4	15391065	1.30E-07
BMSK_chr22	6	18374771	1.09E-07
BMSK_chr23	4	21411059	9.34E-08
BMSK_chr24	5	19152483	1.04E-07
BMSK_chr25	5	15122178	1.32E-07
BMSK_chr26	4	11692837	1.71E-07
BMSK_chr27	1	11124593	1.80E-07
BMSK_chr28	4	10763069	1.86E-07
